# Chemical Constituents from the Vietnamese Mangrove *Avicennia marina*: Two New Iridoid Glycosides and Their Cytotoxicity Against Cancer Cell Lines

**DOI:** 10.3390/ijms26199694

**Published:** 2025-10-05

**Authors:** Ngo Van Hieu, Le Ba Vinh, Pham Thi Mai, Le Ngoc Hung, Nguyen Tien Dat, Lai Ha Phuong, Tran Phương Anh, Do Thanh Tuan, Nguyen Viet Phong, Truong Thi Thu Hien, Hoang Le Tuan Anh

**Affiliations:** 1 Center for High Technology Research and Development, Vietnam Academy of Science and Technology (VAST), 18 Hoang Quoc Viet, Cau Giay, Hanoi 100000, Vietnam; 2 Graduate University of Science and Technology, VAST, 18 Hoang Quoc Viet, Cau Giay, Hanoi 100000, Vietnam; 3 Faculty of Medicine and Pharmacy, Yersin University of Da Lat, Lamdong 670000, Vietnam; vinhrooney@gmail.com; 4 Faculty of Science and Technology, University of Bergen, 5007 Bergen, Norway; 5 Institute for Biotechnology and Environment, NhaTrang University, Northern Nha Trang Ward, Khanh Hoa 57000, Vietnam; 6 Department of Education Quality Assurance and Testing, Thai Binh University of Medicine and Pharmacy, Hung Yen 160000, Vietnam; 7 Department of Biology Education, Teachers College and Institute for Phylogenomics and Evolution, Kyungpook National University, Daegu 41566, Republic of Korea; 8 Center for Training, Research in Toxicology and Radiology, Vietnam Military Medical University, Ha Dong Ward, Hanoi 10000, Vietnam

**Keywords:** mangrove plant, *Avicennia marina*, iridoid glycoside, avicenosides A and B, secondary metabolite, cytotoxicity

## Abstract

*Avicennia marina*, commonly known as the grey mangrove, is a salt-tolerant species widely distributed in coastal and estuarine ecosystems. Traditionally, it has been used in folk medicine to treat skin diseases, rheumatism, and ulcers due to its anti-inflammatory and antimicrobial properties. However, comprehensive studies on the chemical constituents and their pharmacological effects remain limited. The dried powder of the aerial parts of *A. marina* (3.6 kg) was successfully extracted three times with methanol (20 L × 3, each for 2 h) using a multifunctional ultrasonic cleaner operated at 25 °C with a 50% amplitude setting. In this study, the methanolic extract of the aerial parts of *A. marina* led to the isolation of eight compounds, including two previously unreported iridoid glycosides—avicenosides A and B (**1** and **2**)—and six known compounds: techtochrysin (**3**), 7,4′-di-O-methyl-apigenin (**4**), luteolin (**5**), kaempferol (**6**), *trans*-caffeic acid (**7**), and 3,4-dihydroxybenzoic acid (**8**). Their chemical structures were elucidated using nuclear magnetic resonance (NMR) spectroscopy and high-resolution electrospray ionization mass spectrometry (HR-ESI-MS) and compared with previously published data. Moreover, the absolute configuration of the sugar moieties in the new compounds was also identified. All isolated compounds were evaluated for their cytotoxicity against HepG2 and A549 cancer cell lines. The results indicate potential cytotoxicity of the secondary metabolites from *A. marina* and provide evidence of their promising role as lead compounds for the development of novel anticancer agents.

## 1. Introduction

Mangrove forests are vital coastal ecosystems distributed across tropical and subtropical regions, playing essential roles in shoreline protection, carbon sequestration, and supporting high levels of biodiversity [1]. Plants inhabiting these saline and waterlogged environments have developed unique physiological and biochemical adaptations, often resulting in the production of diverse secondary metabolites [2]. These natural compounds have shown a wide range of biological activities, including antimicrobial, anti-inflammatory, antioxidant, and anticancer properties [3]. In recent decades, mangrove species have received increasing scientific attention as promising sources of novel bioactive substances for pharmaceutical development [4]. However, many mangrove plants remain underexplored in terms of their phytochemical composition and pharmacological potential.

Vietnam possesses one of the most extensive mangrove forest systems in Southeast Asia, stretching across coastal provinces from the north to the Mekong Delta in the south [5]. According to recent statistics, as of 2020, Vietnam had approximately 150,000 hectares of mangrove forests, encompassing more than 70 identified mangrove plant species [6]. These ecosystems not only play vital ecological roles—such as coastal protection, carbon sequestration, and supporting biodiversity—but also serve as a rich source of traditional medicinal knowledge [7]. Notably, a large proportion of mangrove plant species in Vietnam remains underexplored in terms of their phytochemical profiles and pharmacological activities. While some preliminary ethnobotanical uses have been documented, systematic investigations into their bioactive compounds and therapeutic mechanisms are still limited. This presents a valuable opportunity for scientific exploration, particularly in the search for novel natural products with potential applications in drug development, especially for cancer, inflammation, and infectious diseases [3,8].

*Avicennia marina* (Vietnamese name: Mắm ổi or Mắm đen) is a mangrove plant widely distributed along subtropical and tropical coastlines [9]. In traditional Egyptian medicine, this plant has been used for the treatment of skin diseases. In Vietnamese traditional medicine, various parts of *A. marina* are also used for managing ailments such as rheumatism, ulcers, and infections [10]. Previous studies have indicated that its crude extracts exhibit several biological activities, including cytotoxic, antioxidant, antimicrobial, and antimalarial effects [10,11]. Phytochemical investigations have revealed a diverse array of constituents, including iridoid glucosides, flavonoids, hydrocarbons, naphthoquinone derivatives, and triterpenes [4]. These findings suggest that *A. marina* is a valuable source of bioactive secondary metabolites with potential pharmacological applications. As a continuation of our ongoing research project aiming to discover potential bioactive agents from Vietnamese mangrove plants [12,13,14,15], this study reports the isolation and structural elucidation of eight compounds from the aerial parts of *A. marina*, including two previously undescribed iridoid glycosides (**1** and **2**) and six known compounds (**3**–**8**). All isolated compounds were also evaluated for their cytotoxic activity.

## 2. Results and Discussion

**Figure 1:**
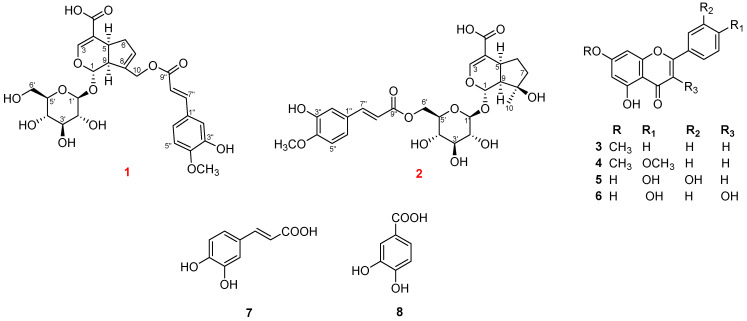
The isolated compounds (**1**–**8**) from *A. marina* included two previously unreported iridoid glycosides—avicenosides A and B (compounds **1** and **2**, highlighted in red). Compounds **3**–**8** were identified as known compounds.

The dried powder of the aerial parts of *A. marina* (3.6 kg) was extracted with methanol (MeOH) (20 L × 3, 2 h each) using a multifunctional ultrasonic cleaner. The MeOH extract was then concentrated and successively partitioned with dichloromethane (CH_2_Cl_2_) and ethyl acetate (EtOAc), yielding the corresponding fractions. Combined chromatographic separation techniques applied to the EtOAc fraction led to the isolation of eight compounds, including two new iridoid glycosides (**1** and **2**) and six known compounds (**3**–**8**) (Figure 1).

**Figure 2:**
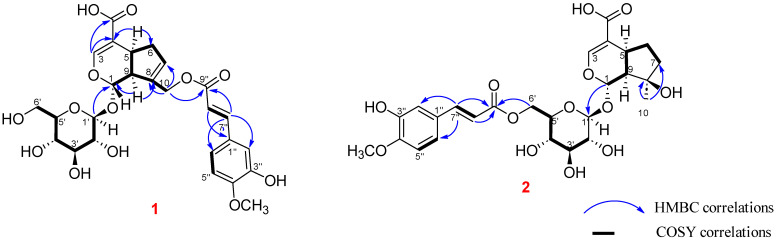
Key ^1^H ^13^C HMBC interactions of compounds **1** and **2**.

**Figure 3:**
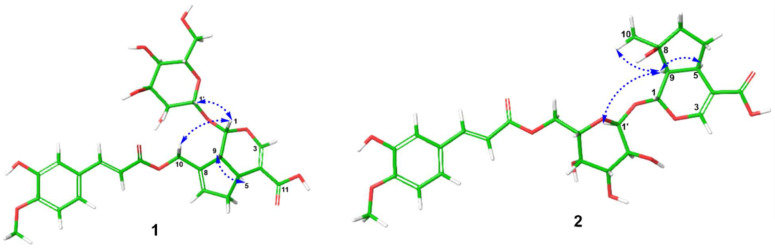
Key ROESY correlations of compounds **1** and **2**. The energy-minimized 3D structures of **1** and **2** were generated using the MMFF force field in Maestro (Schrödinger; Maestro Version 12.5.139, MMshare Version 5.1.139, Release 2020-3; Windows-x64 platform).

Compound **1** was obtained as a brown oil; its molecular formula was identified as C_26_H_30_O_13_ by using HR-ESI-MS, which showed a sodium ion peak at *m*/*z* 573.1577 (calcd for C_26_H_30_O_13_ Na^+^ 573.1579). The ^1^H NMR of **1** showed three aromatic proton signals [7.32 (d, *J* = 1.8 Hz, H-2″), 6.79 (d, *J* = 7.8 Hz, H-5″) and 7.12 (d, *J* = 1.8, 7.8 Hz, H-2″)], four olefinic protons [*δ*_H_ ppm 7.43 (s, H-3), 5.89 (brs, H-3), 7.56 (d, *J* = 16.2 Hz, H-7″) and 6.48 (d, *J* = 16.2 Hz, H-8″)], one hemiacetal proton [5.08 (d, *J* = 7.2 Hz, H-1)], an oxymethylene (4.80, s, H-10), one methylene group [2.10 (m, H-6a) and 2.77 (m, H-6b)], two methine protonic signals [3.08 (m, H-5) and 2.71 (m, H-9)], and an anomeric proton of the glucose unit [4.56 (d, *J* = 7.8 Hz, H-1′)] (Table 1). The ^13^C NMR combined with ^1^H ^13^C HSQC-NMR of compound **1** exhibited 26 carbon signals consisting of ten carbons of the iridoid aglycone [96.1 (C-1), 150.9 (C-3), 111.7 (C-4), 34.9 (C-5), 38.6 (C-6), 129.7 (C-7), 138.2 (C-8), 45.8 (C-9), 61.8 (C-10) and 168.1 (C-11)], ten carbon signals of the *trans*-feruloyl moiety [125.6 (C-1″), 111.2 (C-2″), 149.4 (C-3″), 147.9 (C-4″), 115.5 (C-5″), 123.2 (C-6″), 145.2 (C-7″), 114.3 (C-8″), 166.3 (C-9″) and 55.7 (4″-methoxy)], together with the characteristic signals of the glucose unit [98.8 (C-1′), 73.3 (C-2′), 76.6 (C-3′), 70.2 (C-4′), 77.3 (C-5′) and 61.2 (C-6′)]. The NMR spectrum of **1** is very similar to that of published data of 10-*O*-*E*-caffeoylgeniposidic acid, except for the existence of the *trans*-feruloyl unit in **1** [16]. The sugar moiety was determined as *β*-ᴅ-glucopyranose depending on the large coupling constant J_H-1′/H-2′_ = 7.8 Hz and comparing with the reference [17]. Additionally, the sugar moiety of compound **1** was hydrolyzed with 10% HCl in ethanol at 60 °C overnight. The resulting hydrolysate was subjected to thin-layer chromatography and optical rotation measurement. By comparison with authentic sugar standards, the sugar was identified as D-glucose based on its R_f_ value [R_f_ = 0.30] and its specific optical rotation [[α]ᴅ = +48.2 (c 0.1, H_2_O)], thereby establishing its absolute configuration. The planar chemical structure of **1** was continuously confirmed through the HMBC correlations between H-1′ to C-1 and H-1 to C-1′, suggesting the glucosyl unit was located at C-1 of the aglycone part; H-10 to C-7/C-8/C-9/C-9″, H-7″ to C-1″/C-2″/C-6″/C-8″/C-9″, and H-8″ to C-1″/C-7″/C-9″ to recommend the *trans*-feruloyl group was attached at C-10 (Figure 2). The chemical structure of compound **1** was further determined via the COSY correlations. The COSY data indicated the interactions of H-1/H-9/H-5/H-6/H-7, H-5″/H-6″, H-7″/H-8″ and H-1′/H-2′/H-3′/H-4′/H-5′/H-6′. The relative configuration of compound **1** was determined based on the ROESY spectrum and compared to the chemical shift in the reference [18], interactions between H-5 to H-9, H-1 to H-10, H-1′ to H-9, and compared, suggesting the ring fusion of **1** was in *cis*-form (Figure 3). From the above evidence, compound **1** was determined as a new compound, 10-*O*-*trans*-feruloyl-geniposidic acid, named avicenoside A (Figures S1–S7).

Compound **2** was afforded as a brown oil. The NMR data of **2** showed the characteristic signals of an iridoid glucoside. The ^1^H and HSQC-NMR of **2** presented the signals of the *trans*-feruloyl unit including three protonic signals of the aromatic ABX-substituted system [7.30 (d, *J* = 1.8 Hz, H-2″), 6.78 (d, *J* = 7.8 Hz, H-5″) and 7.11 (dd, *J* = 1.8, 7.8 Hz, H-6″)], one methoxy group [3.81 (s, 4″-methoxy)], two olefinic protons of the *trans*-double bond based on the large coupling constants [7.56 (d, *J* = 16.2 Hz, H-7″) and 6.47 (d, *J* = 16.2 Hz, H-8″)]; together with the signals of the mussaenosidic acid unit consisting of a hemiacetal proton [5.14 (d, *J* = 5.4 Hz, H-1)], an olefinic proton [7.30 (overlapped, H-3)], two methine groups [3.00 (overlapped, H-5) and 1.97 (m, H-9)], one methyl group [1.16 (s, H-10)], two methylene groups [1.22 (m, H-6a), 2.09 (m, H-6b) and 1.52 (m, H-7)], an anomeric proton of the glucosyl unit [4.57 (1H, d, J = 8.4 Hz, H-1′)] and the characteristic protonic signals of the sugar moiety [3.00 (overlapped, H-2′), 3.45 (m, H-3′), 3.18 (m, H-4′), 3.20 (m, H-5′), 4.22 (m, H-6′a) and 4.40 (m, H-6′b)] (Table 1). The ^13^C and HSQC-NMR of **2** presented twenty-six carbon signals to assigned to ten carbons of the aglycone portion of the mussaenosidic acid unit [94.0 (C-1), 149.8 (C-3), 112.0 (C-4), 31.1 (C-5), 29.5 (C-6), 38.8 (C-7), 78.4 (C-8), 50.4 (C-9), 24.5 (C-10) and 167.8 (C-11)], ten carbon signals of the *trans*-feruloyl unit [125.5 (C-1″), 111.0 (C-2″), 149.3 (C-3″), 147.9 (C-4″), 115.4 (C-5″), 123.2 (C-6″), 145.1 (C-7″), 114.3 (C-8″), 166.5 (C-9″) and 55.7 (4″-methoxy)] and six carbon signals of the glucosyl unit [98.3 (C-1′), 73.0 (C-2′), 73.8 (C-3′), 70.1 (C-4′), 76.5 (C-5′) and 63.1 (C-6′)]. The NMR spectral data of **2** is similar to compound marinoid A [18], except for the appearance of the *trans*-feruloyl unit. The HMBC correlations showed the relationships between H-7″ to C-1″/C-2″/C-6″/8″/C-9″, H-8″ to C-1″/C-7″/C-6′, and H-6′ to C-4′/C-5′/C-9″ suggesting the *trans*-feruloyl unit was located at C-6′ of the glucose unit (Figure 2). The ROESY spectrum of compound **2** indicated the interactions of Me-10/H-9, H-9/H-5, H-9/H-6*β*, and H-9/H-1′ to determine the relative configuration of **2** as *cis*-ring fusion (Figure 2). The chemical structure of compound **2** was continuously confirmed via the COSY interactions H-1/H-9/H-5/H-6/H-7, H-1′/H-2′/H-3′/H-4′/H-5′/H-6′, H-5″/H-6″ and H-7″/H-8″. From the above evidence, compound **2** was determined to be 6′-*O*-*trans*-feruloyl-mussaenosidic acid, namely avicenoside B (Figures S8–S14).

Using the same structure elucidation methods, these compounds were identified as six known compounds: techtochrysin (**3**), 7,4′-di-O-methyl-apigenin (**4**), luteolin (**5**), kaempferol (**6**), *trans*-caffeic acid (**7**), and 3,4-dihydroxybenzoic acid (**8**).

**Figure 4:**
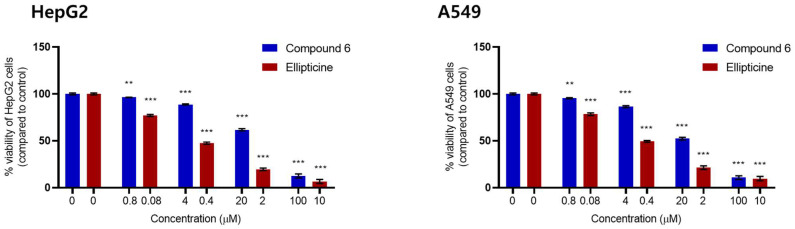
Cytotoxicity of compound **6** and the positive control, ellipticine, tested in a dose-dependent manner in HepG2 and A549 cell lines. Data are presented as the mean ± SD of three independent experiments. ** *p* < 0.01 vs. control group; *** *p* < 0.0001 vs. control group.

Extracts and isolated compounds from Vietnamese mangrove species have exhibited cytotoxicity against several cancer cell lines [8,12,13]. Therefore, ongoing research focuses on investigating the cytotoxic potential and anticancer effects of these compound libraries, aiming to discover novel therapeutic agents from natural sources. Mangrove plants, due to their unique environmental adaptations, produce a diverse array of bioactive secondary metabolites that may offer promising leads in cancer treatment [14,15]. In this study, compounds were isolated from the aerial parts of *A. marina* and subsequently evaluated. All isolated compounds were evaluated for their cytotoxic activity against human hepatocellular carcinoma (HepG2) and lung carcinoma (A549) cell lines at concentrations of 0.8, 4, 20, and 100 μM. The results revealed that compound **6** exhibited an IC_50_ value of 24.14 ± 1.70 μM against HepG2 cells and showed stronger cytotoxicity against A549 cells with an IC_50_ of 20.76 ± 1.35 μM compared to the positive control, ellipticine (Figure S15). The detailed inhibitory concentrations of compound **6** and the positive control, ellipticine, against the two cancer cell lines are presented in Figure 4. These findings suggest a moderate promising anticancer potential of flavonoids derived from *A. marina*. The findings contribute to expanding the chemical and pharmacological knowledge of Vietnamese mangrove flora and highlight their potential as sources of anticancer agents.

Nowadays, natural products play a crucial role in cancer therapy, as a significant proportion of clinically used anticancer drugs are either directly derived from natural sources or synthetically modified analogues of natural compounds [19,20]. These bioactive molecules originate from diverse organisms, including plants, microorganisms, and marine animals, offering unique chemical scaffolds and mechanisms of action [21,22]. According to recent comprehensive reviews and updated statistics, between 1981 and 2019, approximately 49% of all new anticancer agents approved by the FDA were either natural products, their derivatives, or mimetics, highlighting the enduring importance of nature as a prolific source for drug discovery [23]. Natural products are highly valued not only for their potent biological activities but also for their favourable safety profiles and cost-effectiveness compared to many synthetic drugs. Furthermore, the structural diversity and complexity of natural compounds provide unparalleled opportunities to overcome drug resistance and improve therapeutic outcomes. Therefore, the continuous exploration of bioactive natural products from underexplored ecosystems such as mangrove forests holds great promise for developing new, affordable, and effective anticancer agents. In this paper, we describe the isolation and structural characterization of eight compounds **1**–**8** from the aerial parts of *A. marina*, including two new compounds **1**–**2** and six known ones **3**–**8**. The anticancer potential of each isolate was also evaluated using A549 and HepG2 cell lines. These results contribute to the understanding of cytotoxic inhibitors derived from this species and provide further evidence of the chemical diversity and pharmacological potential of this valuable medicinal plant.

## 3. Materials and Methods

### 3.1. General Experimental Procedures

The NMR (Nuclear Magnetic Resonance) spectra were recorded on a Bruker Avance III 600 MHz spectrometer (Karlsruhe, Germany), using tetramethylsilane (TMS) as an internal standard. The HR-ESI-MS (High-resolution electrospray ionization mass spectrometry) experiments were performed by using a MicrOTOF-Q III mass spectrometer (Bruker Daltonics, Bremen, Germany). Silica gel (Kieselgel 60, 230–400 mesh, Merck, Darmstadt, Germany), Sephadex LH-20 (Sigma-Aldrich, Saint Louis, MO, USA), and RP-18 resins (150 µm, Fuji Silysia Chemical Ltd., Kasugai, Japan) were used for the open column chromatography (CC). Thin-layer chromatography (TLC) was conducted using silica gel and RP-18 plates (Merck KGaA, Darmstadt, Germany). Compounds were visualized by the mixture of solvents sulfuric acid-methanol (1:10, *v*:*v*) and further heated at high temperature for 2–3 min.

### 3.2. Plant Identification

The aerial portions of the *A. marina* were collected in Thai Binh province on 12 December 2024. Its scientific name was further identified by Dr. Do Thanh Tuan (Thai Binh University of Medicine and Pharmacy). Its specimen (AM2022) was deposited at the Centre for High Research and Development, VAST, Hanoi, Vietnam.

### 3.3. Extraction and Isolation

The dried powder of the aerial parts of *Avicennia marina* (3.6 kg) was extracted three times with methanol (20 L × 3, each for 2 h) using a multifunctional ultrasonic cleaner at 25 °C with a 50% amplitude setting. The combined methanolic extracts were concentrated under reduced pressure to yield a crude residue of 362 g. This residue was suspended in hot water (2 L) and successively partitioned with dichloromethane (CH_2_Cl_2_) and ethyl acetate (EtOAc), affording three fractions: the CH_2_Cl_2_ fraction (AMC, 131 g), the EtOAc fraction (AME, 153 g), and the aqueous layer.

The EtOAc fraction (AME, 153 g) was subjected to silica gel column chromatography using a gradient of CH_2_Cl_2_–MeOH (150:1 to 1:1, *v*/*v*), yielding ten subfractions (AME1–AME10). Fraction AME3 (5.0 g) was further purified by Sephadex LH-20 column chromatography with methanol to afford compound **3** (3.0 mg) and subfractions AME3.1–AME3.3. Subfraction AME3.2 (121 mg) was subsequently chromatographed on an RP-18 reversed-phase column with MeOH–H_2_O (3:1, *v*/*v*) to yield compound **4** (5.3 mg).

Fraction AME5 (6.3 g) was subjected to silica gel chromatography with CH_2_Cl_2_–MeOH (20:1, *v*/*v*), giving compound **5** (13 mg) along with subfractions AME5.1–AME5.4. Subfraction AME5.3 (200 mg) was purified by Sephadex LH-20 column chromatography using methanol to obtain compound **6** (7.0 mg) and subfractions AME5.3.1–AME5.3.3. Compounds **7** (6.3 mg) and **8** (3.5 mg) were isolated from subfraction AME5.3.2 (37 mg) via RP-18 column chromatography using MeOH–H_2_O (2:1, *v*/*v*).

Fraction AME7 (8.0 g) was chromatographed over silica gel with EtOAc–MeOH–H_2_O (20:1:1, *v*/*v*/*v*), yielding compound **1** (30 mg) and subfractions AME7.1–AME7.6. Further purification of AME7.3 (300 mg) by Sephadex LH-20 column chromatography with methanol afforded compound **2** (25 mg) along with subfractions AME7.3.1–AME7.3.3.

Avicenoside A (**1**): Brown oil; HR-ESI-MS *m*/*z* 573.1577 [M+Na]^+^ (calcd for C_26_H_30_O_13_Na^+^, 573.1579). ^1^H-NMR (DMSO-*d*_6_, 600 MHz): *δ* 2.10 (1H, m, H-6a), 2.71 (1H, m, H-9), 2.77 (1H, m, H-6b), 3.00 (1H, m, H-2′), 3.05 (1H, m, H-4′), 3.08 (1H, m, H-5), 3.14 (1H, m, H-5′), 3.17 (1H, m, H-3′), 3.40 (1H, m, H-6′a), 3.65 (1H, m, H-6′b), 3.82 (3H, s, 4″-methoxy), 4.56 (1H, d, *J* = 7.8 Hz, H-1′), 4.80 (2H, s, H-10), 5.08 (1H, d, *J* = 7.2 Hz, H-1), 5.89 (1H, brs, H-7), 6.48 (1H, d, *J* = 16.2 Hz, H-8″), 6.79 (1H, d, *J* = 7.8 Hz, H-5″), 7.12 (1H, dd, *J* = 1.8, 7.8 Hz, H-2″), 7.32 (1H, d, *J* = 1.8 Hz, H-2″), 7.43 (1H, s, H-3) and 7.56 (1H, d, *J* = 16.2 Hz, H-7″). ^13^C-NMR (DMSO-*d*_6_, 150 MHz): *δ* 34.9 (C-5), 38.6 (C-6), 45.8 (C-9), 55.7 (C-4″-methoxy), 61.2 (C-6′), 61.8 (C-10), 70.2 (C-4′), 73.3 (C-2′), 76.6 (C-3′), 77.3 (C-5′), 96.1 (C-1), 98.8 (C-1′), 111.2 (C-2″), 111.7 (C-4), 114.3 (C-8″), 115.5 (C-5″), 123.2 (C-6″), 125.6 (C-1″), 129.7 (C-7), 138.2 (C-8), 145.2 (C-7″), 147.9 (C-4″), 149.4 (C-3″), 150.9 (C-3), 166.3 (C-9″) and 168.1 (C-11).

Avicenoside B (**2**): Brown oil; HR-ESI-MS *m*/*z* 575.1736 [M+Na]^+^ (calcd for C_26_H_32_O_13_Na^+^, 575.1735). ^1^H-NMR (DMSO-*d*_6_, 600 MHz): *δ* 1.16 (3H, s, H-10), 1.22 (1H, m, H-6a), 1.52 (2H, m, H-7), 1.97 (1H, m, H-9), 2.09 (1H, m, H-6b), 3.00 (1H, overlapped, H-5), 3.00 (1H, overlapped, H-2′), 3.18 (1H, m, H-4′), 3.20 (1H, m, H-5′), 3.45 (1H, m, H-3′), 3.81 (3H, s, 4″-methoxy), 4.22 (1H, m, H-6′a), 4.40 (1H, m, H-6′b), 4.57 (1H, d, *J* = 8.4 Hz, H-1′), 5.14 (1H, d, *J* = 5.4 Hz, H-1), 6.47 (1H, d, *J* = 16.2 Hz, H-8″), 6.78 (1H, d, *J* = 7.8 Hz, H-5″), 7.11 (1H, dd, *J* = 1.8, 7.8 Hz, H-6″), 7.30 (1H, d, *J* = 1.8 Hz, H-2″) and 7.56 (1H, d, *J* = 16.2 Hz, H-7″). ^13^C-NMR (DMSO-*d*_6_, 150 MHz): *δ* 24.5 (C-10), 29.5 (C-6), 31.1 (C-5), 38.8 (C-7), 50.4 (C-9), 55.7 (4″-methoxy), 63.1 (C-6′), 70.1 (C-4′), 73.0 (C-2′), 73.8 (C-3′), 76.5 (C-5′), 78.4 (C-8), 94.0 (C-1), 98.3 (C-1′), 111.0 (C-2″), 112.0 (C-4), 114.3 (C-8″), 115.4 (C-5″), 123.2 (C-6″), 125.5 (C-1″), 145.1 (C-7″), 147.9 (C-4″), 149.3 (C-3″),149.8 (C-3), 166.5 (C-9″) and 167.8 (C-11).

### 3.4. Acid Hydrolysis and Identification of Absolute Configuration of Sugars

Compounds **1** and **2** (6.5 mg) were subjected to acid hydrolysis by heating with 10% HCl in ethanol at 60 °C overnight. After hydrolysis, the reaction mixture was partitioned with chloroform and water to separate the aglycone. The aqueous layer was then evaporated to dryness under a stream of nitrogen, and the residue was redissolved in water (1 mg/mL) for optical rotation measurement. The optical rotation values were recorded and compared with those of authentic sugar standards. The sugar moiety was identified as *D*-glucose based on its optical rotation and Rf value in thin-layer chromatography [Rf = 0.30, [α]ᴅ = +48.2 (c 0.1, H_2_O)] [24].

### 3.5. Cytotoxicity Assay

The cytotoxic activity of the isolated compounds was evaluated against two human cancer cell lines: A549 (ATCC CCL-185), a non-small cell lung cancer (NSCLC) line, and HepG2 (ATCC HB-8065), a hepatocarcinoma line. These cell lines were kindly provided by Prof. Dr. J. M. Pezzuto (Long Island University, USA) and Prof. Chi-Ying Huang (National Yang Ming Chiao Tung University, Taiwan). Cell viability was assessed using the sulforhodamine B (SRB) colorimetric assay, which quantifies total cellular protein content as an indirect measure of cell number. The assay was conducted according to the method described by Giang, V.H. et al., wherein the optical density (OD) of SRB-stained cellular proteins is measured to estimate cell mass. Cells were exposed to each compound for 72 h at concentrations of 100, 20, 4, and 0.8 μM. Ellipticine served as the positive control at concentrations of 10, 2, 0.4, and 0.08 μg/mL. All experiments were performed in triplicate to ensure reproducibility and statistical reliability. Dimethyl sulfoxide (DMSO) at 1% was used as the negative control, with a final DMSO concentration of 0.05% in each well. Dose–response curves were generated, and half-maximal inhibitory concentration (IC_50_) values were calculated using GraphPad Prism software (version 9.5.1, GraphPad Software, San Diego, CA, USA). According to the U.S. National Cancer Institute (NCI) criteria, a crude extract is considered to exhibit significant cytotoxic activity if its IC_50_ value is ≤20 μg/mL, while a pure compound with an IC_50_ ≤ 5 μM is generally regarded as demonstrating outstanding activity with potential relevance for anticancer drug development.

## 4. Conclusions

As part of our ongoing investigation of Vietnamese mangrove forests aimed at exploring structurally diverse bioactive compounds, a series of metabolites with significant anticancer and anti-inflammatory potential has been identified. These compounds include triterpenoid saponins, diterpenoids, triterpenoids, phenolics, and other related classes, highlighting the chemical richness and pharmacological promise of mangrove-derived natural products [8,12,13,14]. The MeOH extract of the aerial parts of *A. marina* led to the isolation of eight compounds including two previously unreported iridoid glycosides—avicenosides A and B (**1** and **2**)—and six known compounds: techtochrysin (**3**), 7,4′-di-O-methyl-apigenin (**4**), luteolin (**5**), kaempferol (**6**), *trans*-caffeic acid (**7**), and 3,4-dihydroxybenzoic acid (**8**). Their chemical structures were elucidated using nuclear magnetic resonance (NMR) spectroscopy and high-resolution electrospray ionization mass spectrometry (HR-ESI-MS) and compared with previously published data. Compound **6** displayed an IC_50_ of 24.14 ± 1.70 μM against HepG2 cells and exhibited even greater cytotoxicity against A549 cells, with an IC_50_ of 20.76 ± 1.35 μM, surpassing that of the positive control, ellipticine. Although in vivo evaluation is still needed, the results reveal the cytotoxic potential of *A. marina* secondary metabolites and their promise as lead compounds for novel anticancer agents, highlighting the value of Vietnamese mangrove plants and opening avenues for future research on their bioactive compounds and pharmacological applications.

## Figures and Tables

**Table 1:** The NMR data of compounds **1** and **2** (DMSO-*d*_6_).

Position	*δ*_C_ ^a^	*δ*_H_ ^b^ (mult., *J* in Hz)	*δ*_C_ ^a^	*δ*_H_ ^b^ (mult., *J* in Hz)
1	96.1	5.08 (1H, d, *J* = 7.2 Hz, H-1)	94.0	5.14 (1H, d, *J* = 5.4 Hz, H-1)
3	150.9	7.43 (1H, s, H-3)	149.8	7.30 (1H, overlapped, H-3)
4	111.7	-	112.0	-
5	34.9	3.08 (1H, m, H-5)	31.1	3.00 (1H, overlapped, H-5)
6	38.6	2.10 (1H, m, H-6a) 2.77 (1H, m, H-6b)	29.5	1.22 (1H, m, H-6a) 2.09 (1H, m, H-6b)
7	129.7	5.89 (1H, brs, H-7)	38.8	1.52 (2H, m, H-7)
8	138.2	-	78.4	-
9	45.8	2.71 (1H, m, H-9)	50.4	1.97 (1H, m, H-9)
10	61.8	4.80 (2H, s, H-10)	24.5	1.16 (3H, s, H-10)
11	168.1	-	167.8	-
1′	98.8	4.56 (1H, d, *J* = 7.8 Hz, H-1′)	98.3	4.57 (1H, d, *J* = 8.4 Hz, H-1′)
2′	73.3	3.00 (1H, m, H-2′)	73.0	3.00 (1H, overlapped, H-2′)
3′	76.6	3.17 (1H, m, H-3′)	73.8	3.45 (1H, m, H-3′)
4′	70.0	3.05 (1H, m, H-4′)	70.1	3.18 (1H, m, H-4′)
5′	77.3	3.14 (1H, m, H-5′)	76.5	3.20 (1H, m, H-5′)
6′	61.2	3.40 (1H, m, H-6′a) 3.65 (1H, m, H-6′b)	63.1	4.22 (1H, m, H-6′a) 4.40 (1H, m, H-6′b)
1″	125.6	-	125.5	-
2″	111.2	7.32 (1H, d, *J* = 1.8 Hz, H-2″)	111.0	7.30 (1H, d, *J* = 1.8 Hz, H-2″)
3″	149.4	-	149.3	-
4″	147.9	-	147.9	-
5″	115.5	6.79 (1H, d, *J* = 7.8 Hz, H-5″)	115.4	6.78 (1H, d, *J* = 7.8 Hz, H-5″)
6″	123.2	7.12 (1H, dd, *J* = 1.8, 7.8 Hz, H-2″)	123.2	7.11 (1H, dd, *J* = 1.8, 7.8 Hz, H-6″)
7″	145.2	7.56 (1H, d, *J* = 16.2 Hz, H-7″)	145.1	7.56 (1H, d, *J* = 16.2 Hz, H-7″)
8″	114.3	6.48 (1H, d, *J* = 16.2 Hz, H-8″)	114.3	6.47 (1H, d, *J* = 16.2 Hz, H-8″)
9″	166.3	-	166.5	-
4″- OCH_3_	55.7	3.82 (3H, s, 4″-OCH_3_)	55.7	3.81 (3H, s, 4″-OCH_3_)

^a^ Recorded at 600 MHz, ^b^ Recorded at 150 MHz. The structural elucidation was performed using ^1^H NMR, ^13^C NMR, ^1^H–^13^C HSQC, ^1^H–^13^C HMBC, ^1^H–^1^H ROESY, and ^1^H–^1^H COSY experiments.

## Data Availability

The data presented in this study is available on request from the corresponding author.

## Supplementary Materials

The following supporting information can be downloaded at: https://www.mdpi.com/article/10.3390/ijms26199694/s1.
