# Supplementary material for: Chemical Constituents from the Vietnamese Mangrove Avicennia marina: Two New Iridoid Glycosides and Their Cytotoxicity Against Cancer Cell Lines
Source: Int J Mol Sci. 2025 Oct 5;26(19):9694. doi: 10.3390/ijms26199694 (PMC12525465; doi:10.3390/ijms26199694)
Supplement: Supplementary file 1 [file ijms-26-09694-s001.zip › ijms-3835005-supplementary.pdf]

Communication

Chemical Constituents from the Vietnamese Mangrove *Avicennia marina*: Two New Iridoid Glycosides and Their Cytotoxicity Against Cancer Cell Lines

Ngo Van Hieu [1,2,†], Le Ba Vinh [3,4,†], Pham Thi Mai [2,5], Le Ngoc Hung [1], Nguyen Tien Dat [1], Lai Ha Phuong [1], Tran Phương Anh [2], Do Thanh Tuan [6], Nguyen Viet Phong [7], Truong Thi Thu Hien [8] and Hoang Le Tuan Anh [1,2,*]

1 Center for High Technology Research and Development, Vietnam Academy of Science and Technology (VAST), 18 Hoang Quoc Viet, Cau Giay, Hanoi 100000, Vietnam
2 Graduate University of Science and Technology, VAST, 18 Hoang Quoc Viet, Cau Giay, Hanoi 100000, Vietnam
3 Faculty of Medicine and Pharmacy, Yersin University of Da Lat, Lamdong 670000, Vietnam; vinhrooney@gmail.com
4 Faculty of Science and Technology, University of Bergen, 5007 Bergen, Norway
5 Institute for Biotechnology and Environment, NhaTrang University, Northern Nha Trang Ward, Khanh Hoa 57000, Vietnam
6 Department of Education Quality Assurance and Testing, Thai Binh University of Medicine and Pharmacy, Hung Yen 160000, Vietnam
7 Department of Biology Education, Teachers College and Institute for Phylogenomics and Evolution, Kyungpook National University, Daegu 41566, Republic of Korea
8 Center for Training, Research in Toxicology and Radiology, Vietnam Military Medical University, Ha Dong Ward, Hanoi 10000, Vietnam
* Correspondence: hltanh@cdtd.vast.vn
† These authors contributed equally to this study.

Abstract

Avicennia marina, commonly known as the grey mangrove, is a salt-tolerant species widely distributed in coastal and estuarine ecosystems. Traditionally, it has been used in folk medicine to treat skin diseases, rheumatism, and ulcers due to its anti-inflammatory and antimicrobial properties. However, comprehensive studies on the chemical constituents and their pharmacological effects remain limited. The dried powder of the aerial parts of *A. marina* (3.6 kg) was successfully extracted three times with methanol (20 L × 3, each for 2 hours) using a multifunctional ultrasonic cleaner operated at 25 °C with a 50% amplitude setting. In this study, the methanolic extract of the aerial parts of *A. marina* led to the isolation of eight compounds, including two previously unreported iridoid glycosides—avicenosides A and B (**1** and **2**)—and six known compounds: techtochrysin (**3**), 7,4'-di-O-methyl-apigenin (**4**), luteolin (**5**), kaempferol (**6**), trans-caffeic acid (**7**), and 3,4-dihydroxybenzoic acid (**8**). Their chemical structures were elucidated using nuclear magnetic resonance (NMR) spectroscopy and high-resolution electrospray ionization mass spectrometry (HR-ESI-MS) and compared with previously published data. Moreover, the absolute configuration of the sugar moieties in the new compounds was also identified. All isolated compounds were evaluated for their cytotoxicity against HepG2 and A549 cancer cell lines. The results indicate potential cytotoxicity of the secondary metabolites from *A. marina* and provided evidence of their promising role as lead compounds for the development of novel anticancer agents.

Keywords: *Avicennia marina*, iridoid glycoside, avicenosides A and B, secondary metabolite, cytotoxicity.

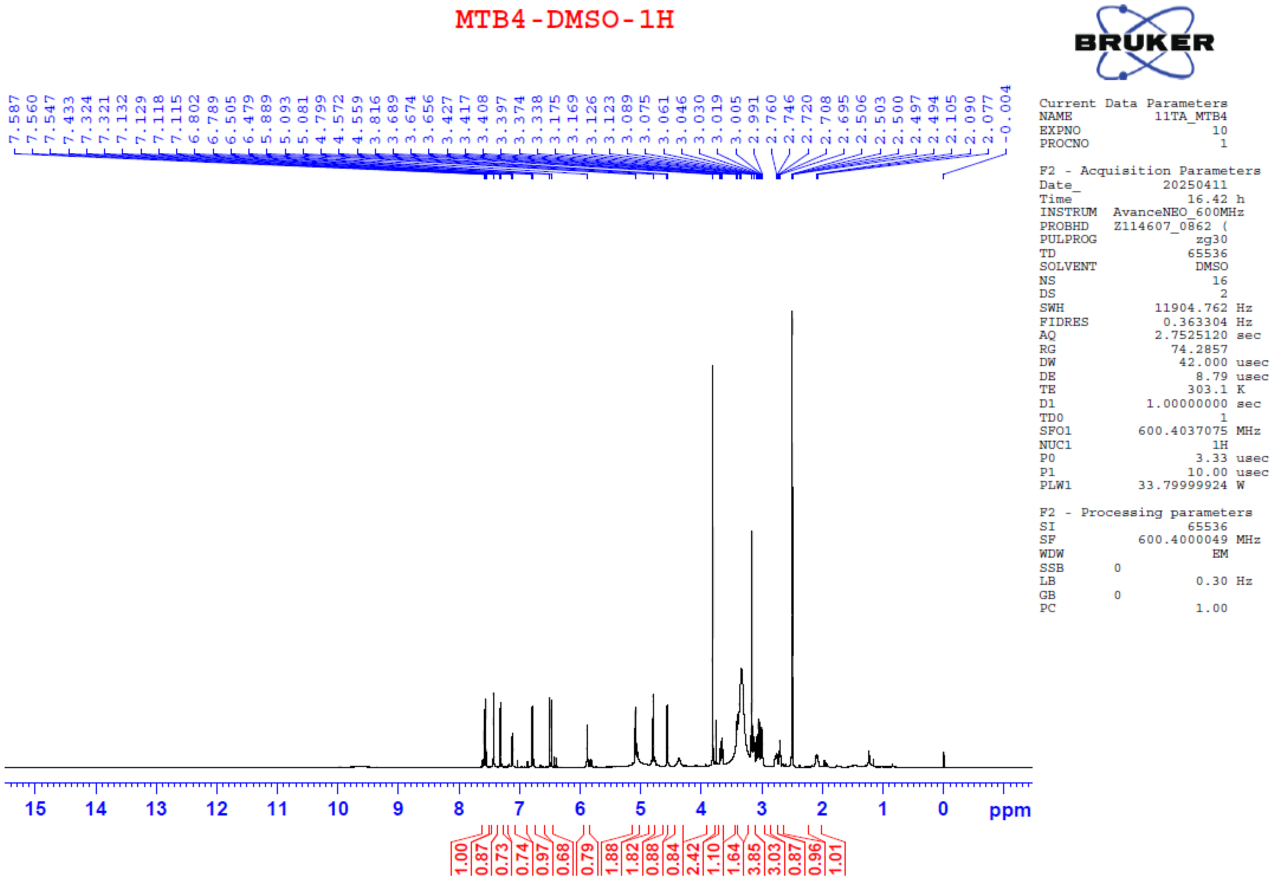

Figure S1. ^{1}H-NMR spectrum of compound **1** and its expanded spectrum

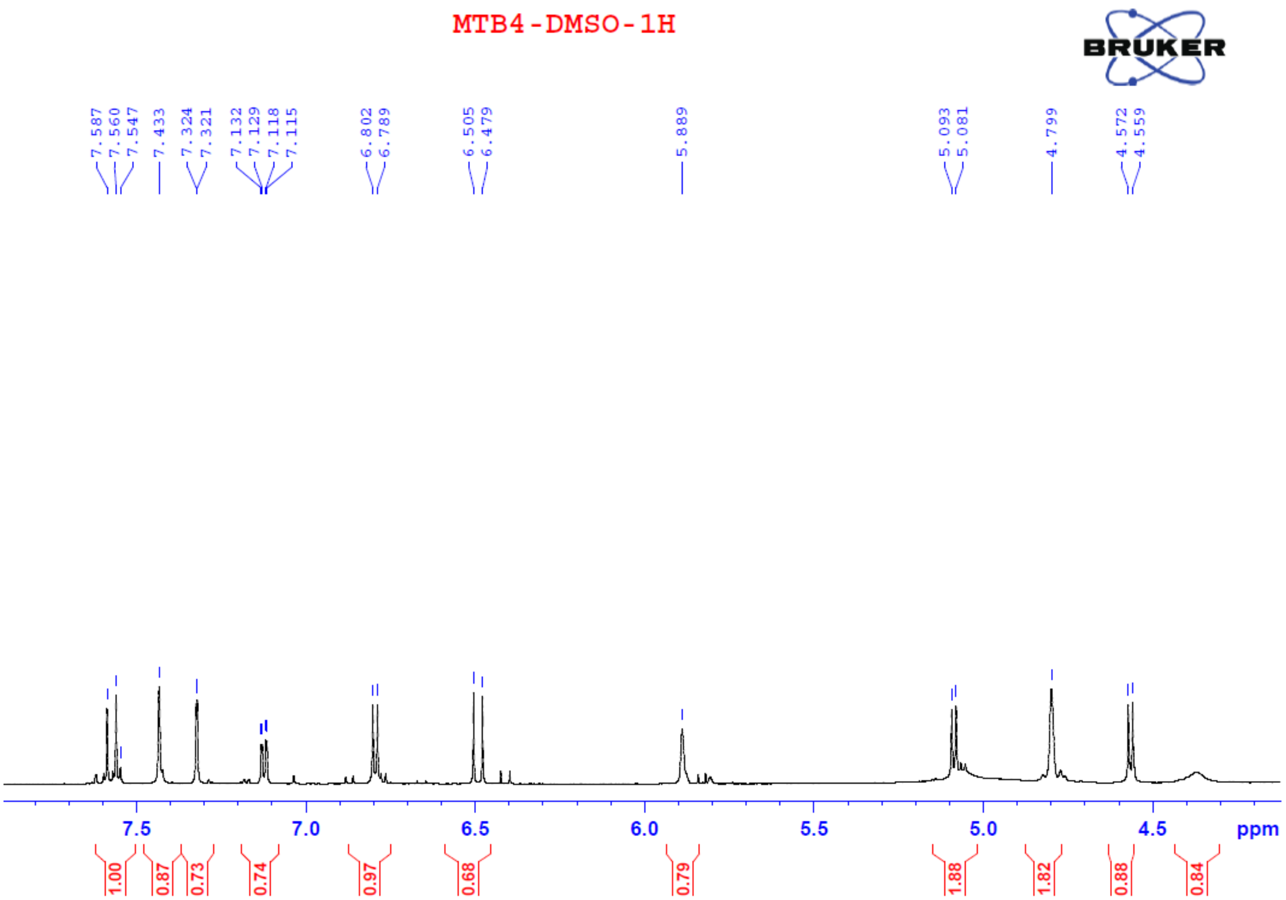
MTB4-DMSO-1H
BRUKER
7.587
7.560
7.547
7.433
7.324
7.321
7.132
7.129
7.118
7.115
6.802
6.789
6.505
6.479
5.889
5.093
5.081
4.799
4.572
4.559
7.5
7.0
6.5
6.0
5.5
5.0
4.5
ppm
1.00
0.87
0.73
0.74
0.97
0.68
0.79
1.88
1.82
0.88
0.84

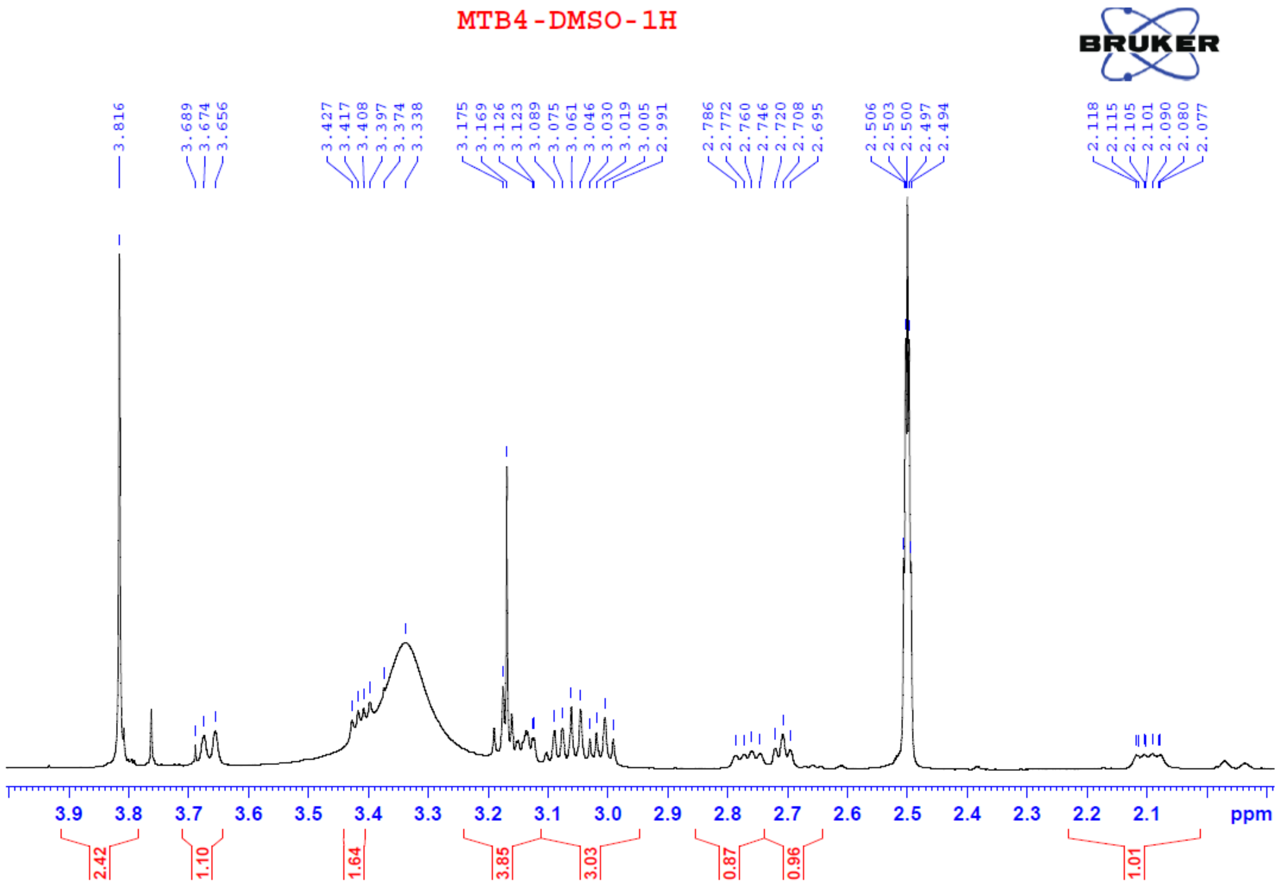
MTB4-DMSO-1H
BRUKER
3.816
3.689
3.674
3.656
3.427
3.417
3.408
3.397
3.374
3.338
3.175
3.169
3.126
3.123
3.089
3.075
3.061
3.046
3.030
3.019
3.005
2.991
2.786
2.772
2.760
2.746
2.720
2.708
2.695
2.506
2.503
2.500
2.497
2.494
2.118
2.115
2.105
2.101
2.090
2.080
2.077
3.9
3.8
3.7
3.6
3.5
3.4
3.3
3.2
3.1
3.0
2.9
2.8
2.7
2.6
2.5
2.4
2.3
2.2
2.1
ppm
2.42
1.10
1.64
3.85
3.03
0.87
0.96
1.01

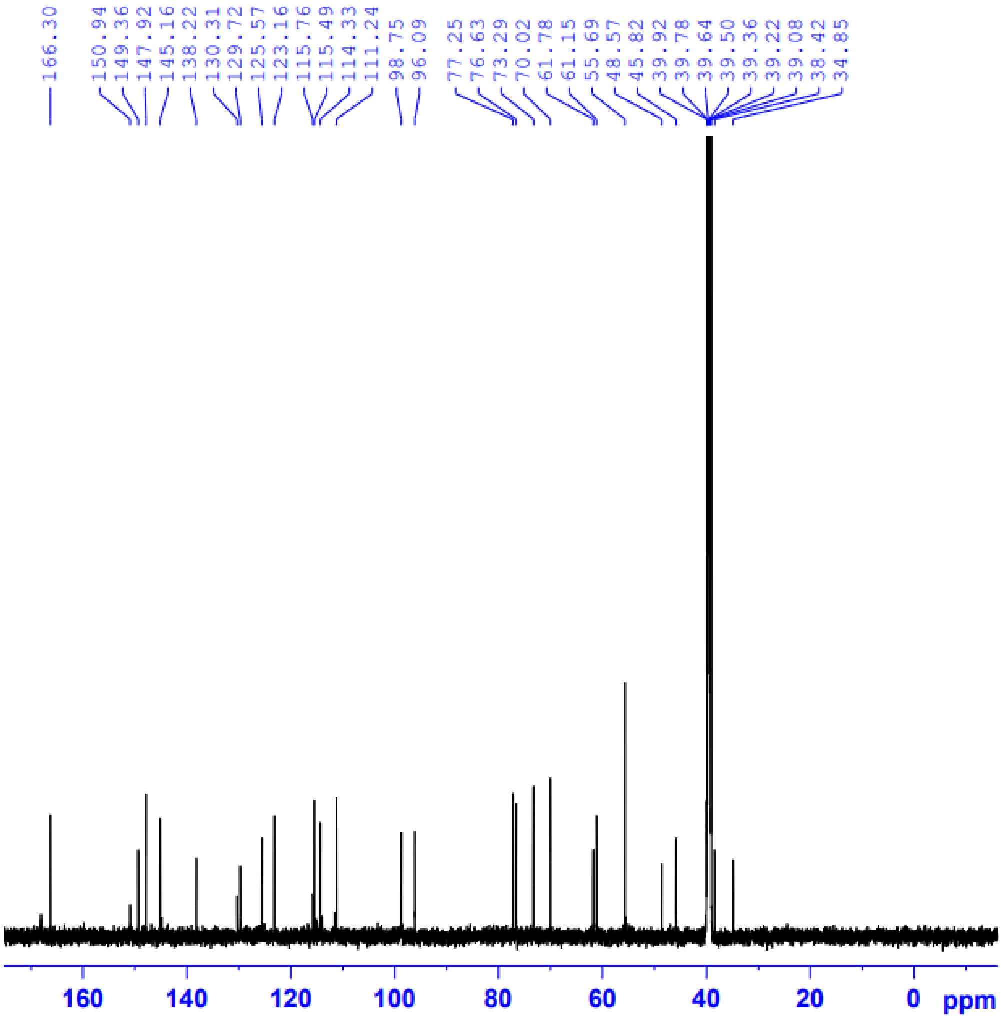

Figure S2. ^{13}C-NMR spectrum of compound **1** and its expanded spectrum

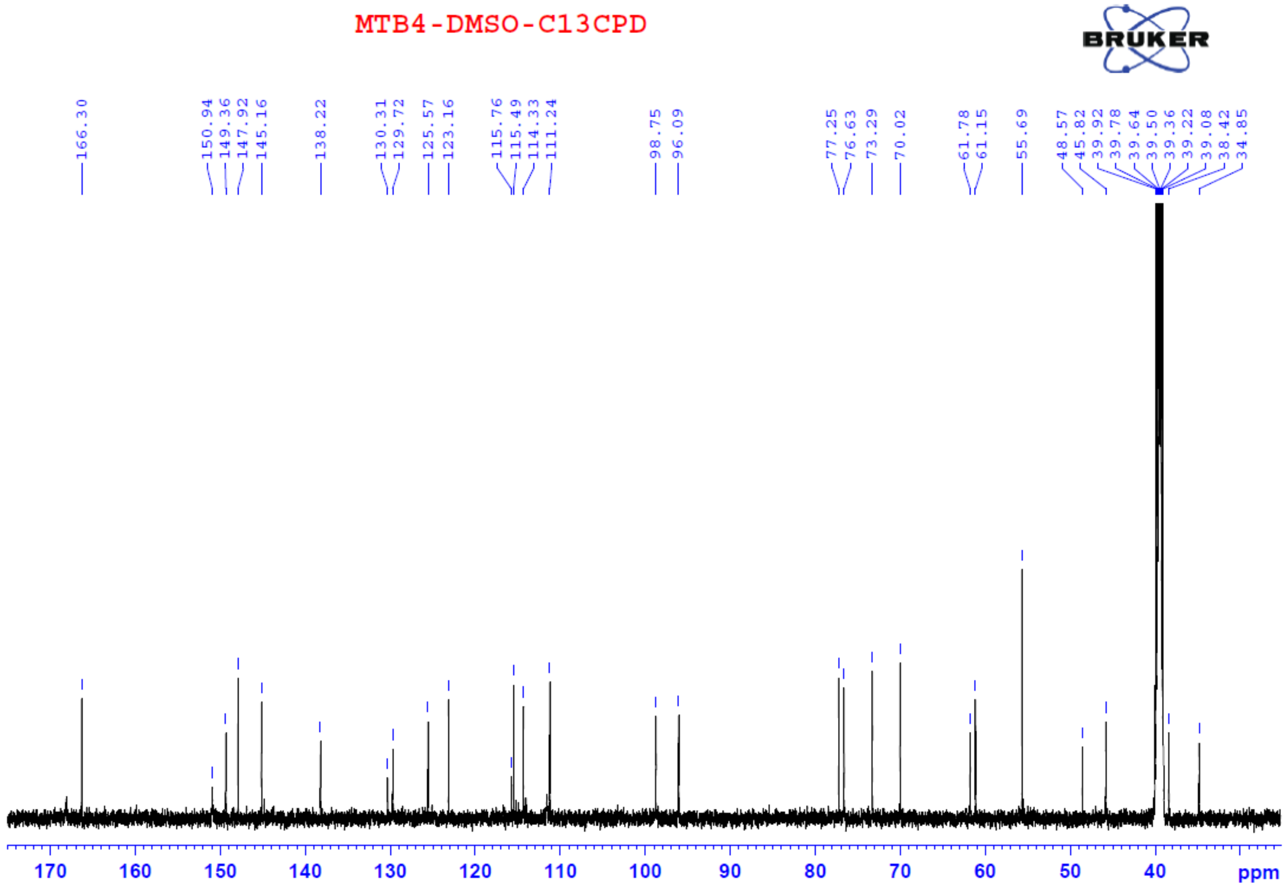
MTB4-DMSO-C13CPD
BRUKER
166.30
150.94
149.36
147.92
145.16
138.22
130.31
129.72
125.57
123.16
115.76
115.49
114.33
111.24
98.75
96.09
77.25
76.63
73.29
70.02
61.78
61.15
55.69
48.57
45.82
39.92
39.78
39.64
39.50
39.36
39.22
39.08
38.42
34.85
170 160 150 140 130 120 110 100 90 80 70 60 50 40 ppm

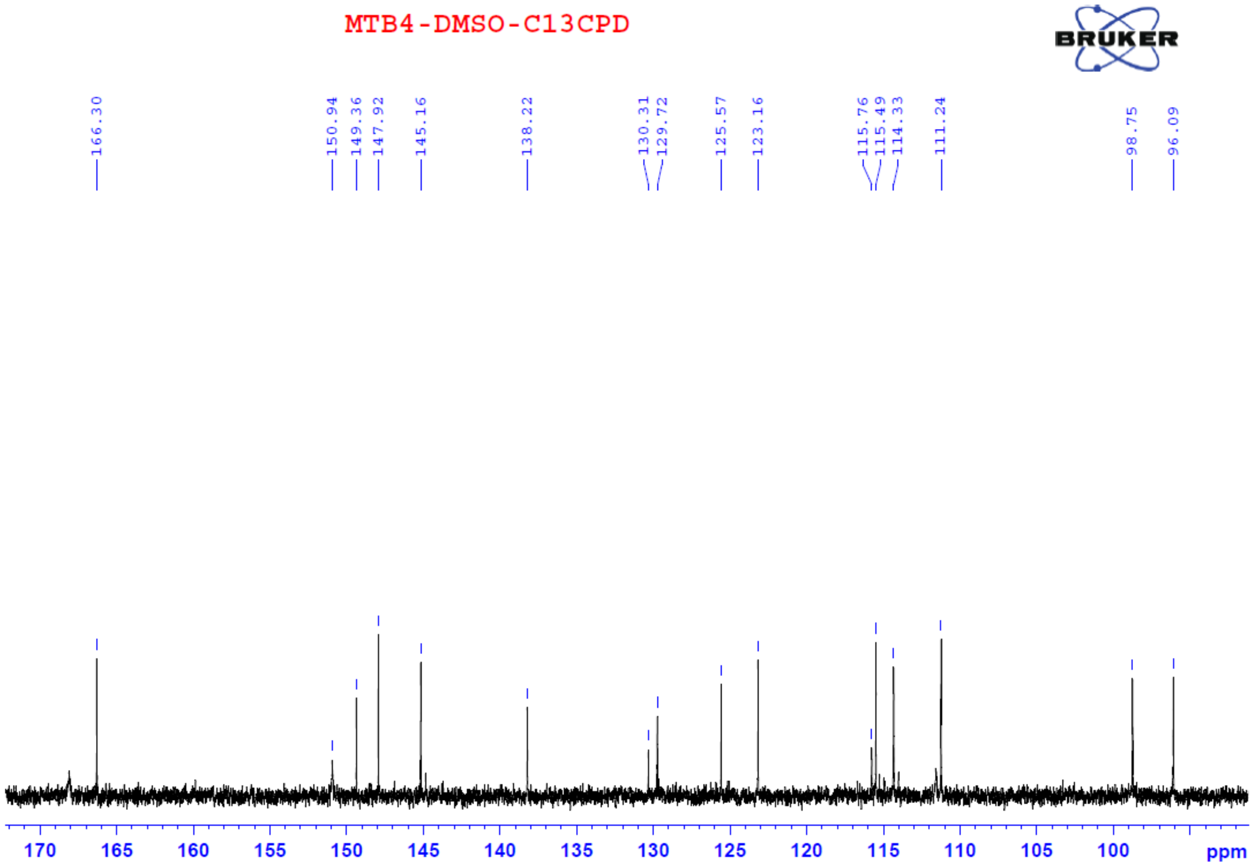
MTB4-DMSO-C13CPD
BRUKER
166.30
150.94
149.36
147.92
145.16
138.22
130.31
129.72
125.57
123.16
115.76
115.49
114.33
111.24
98.75
96.09
170 165 160 155 150 145 140 135 130 125 120 115 110 105 100 ppm

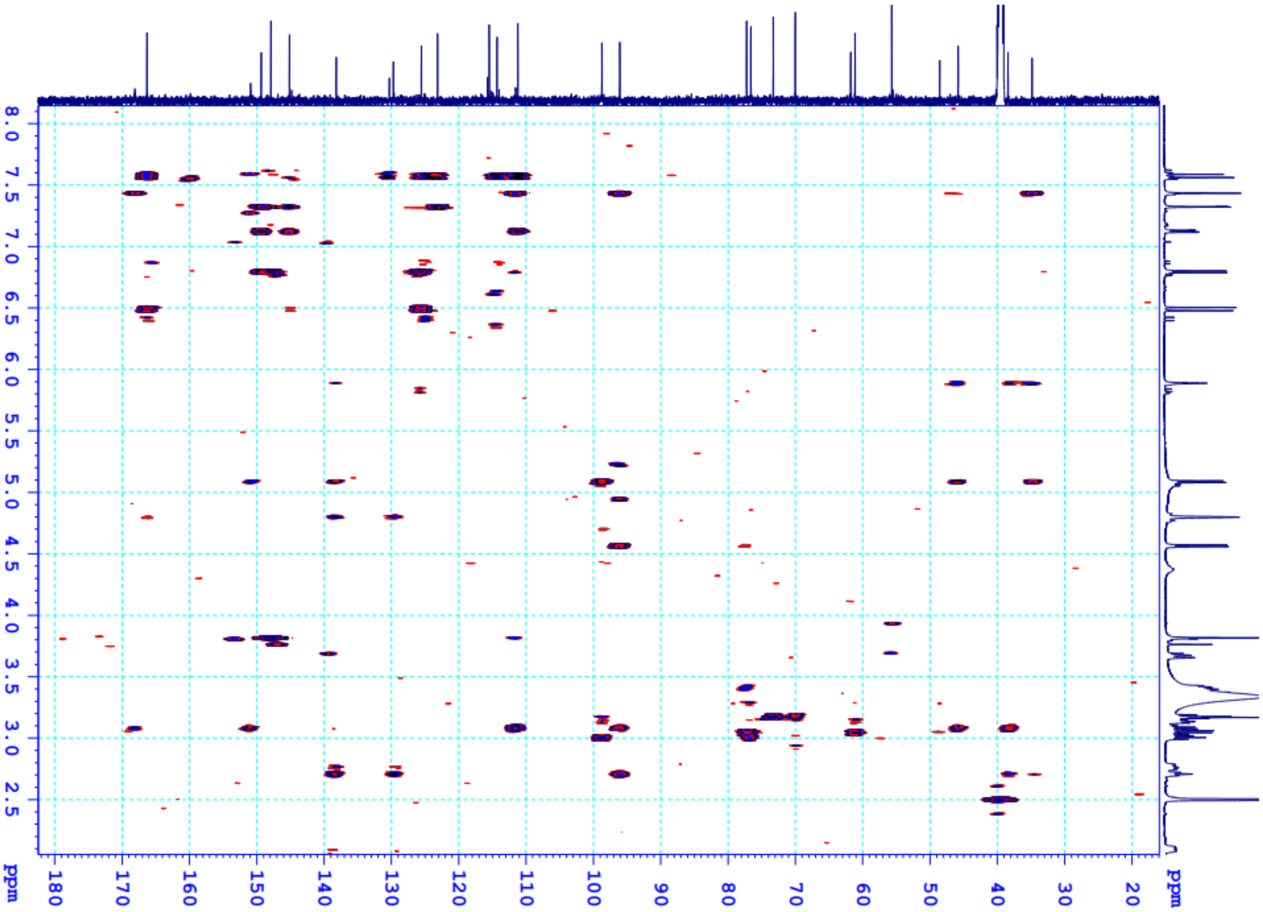

Figure S3. HMBC spectrum of compound **1**

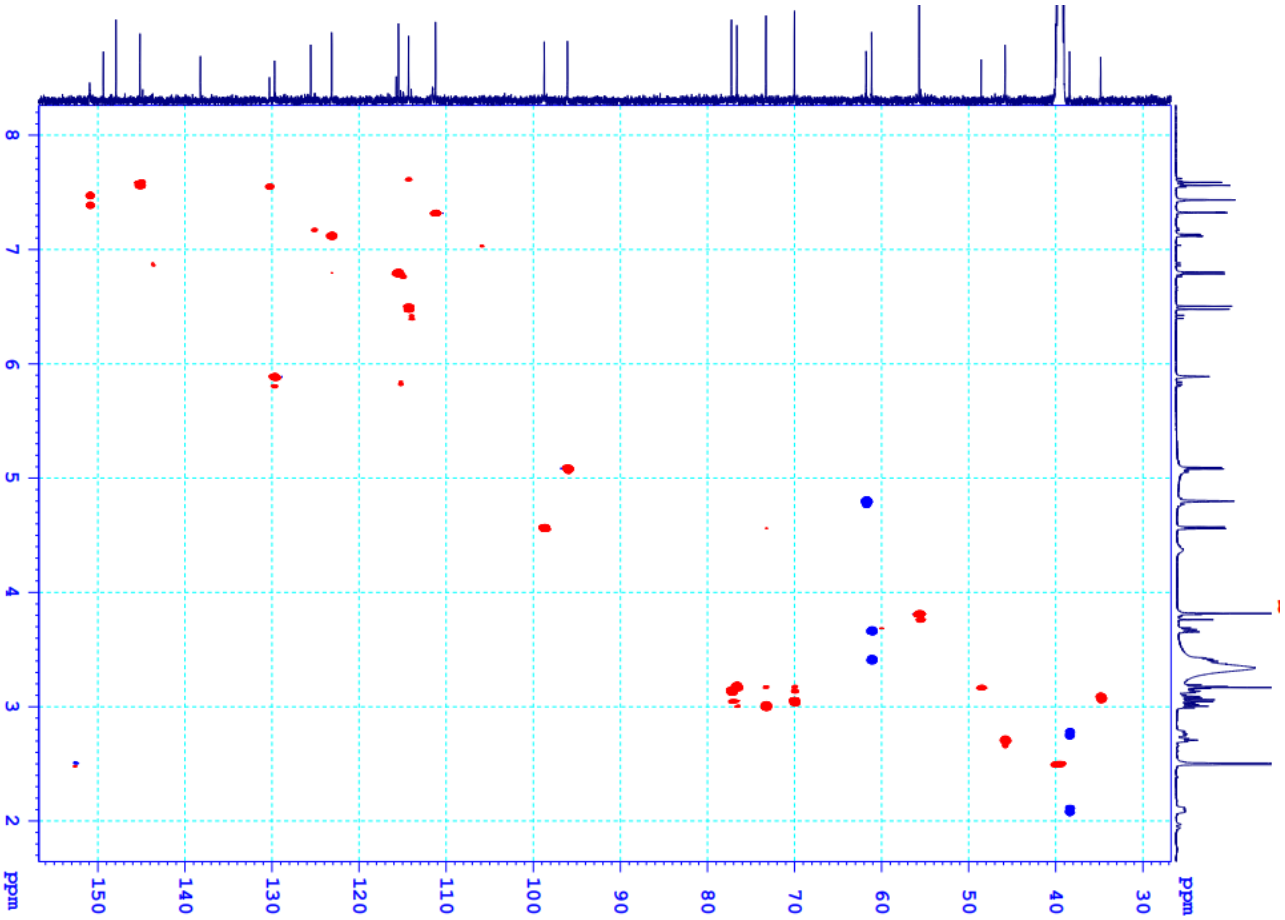

Figure S4. HSQC spectrum of compound **1**

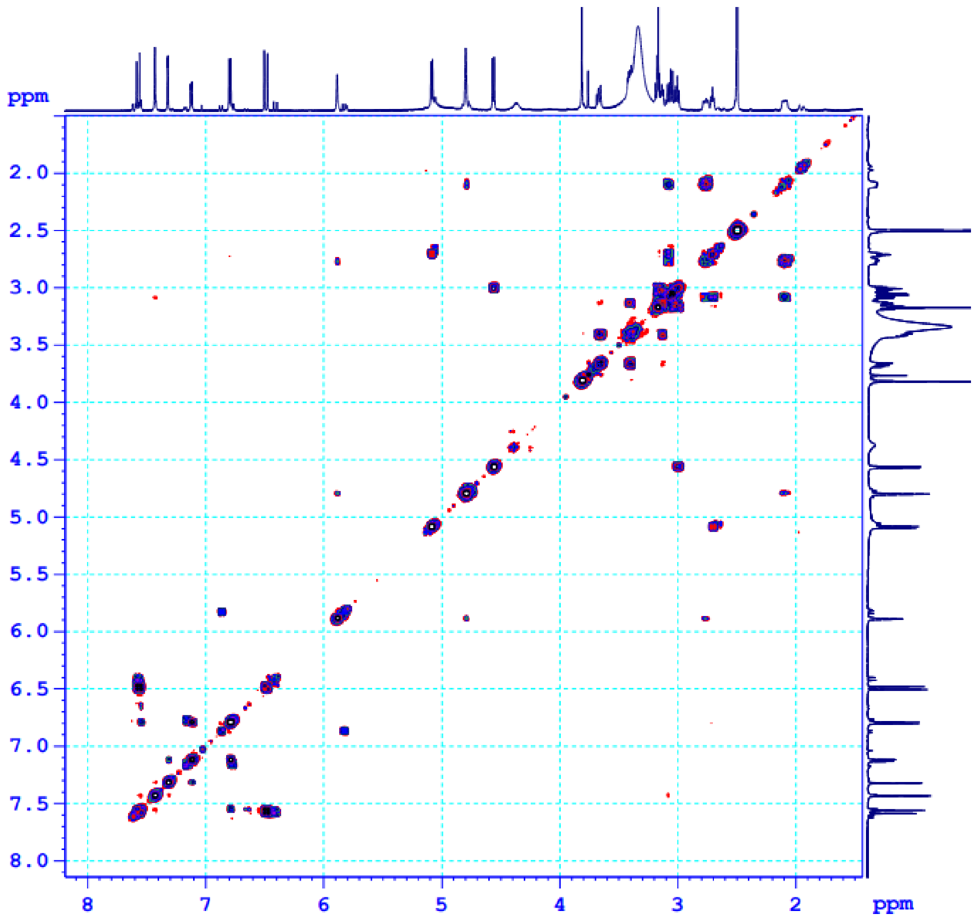

Figure S5. COSY spectrum of compound **1**

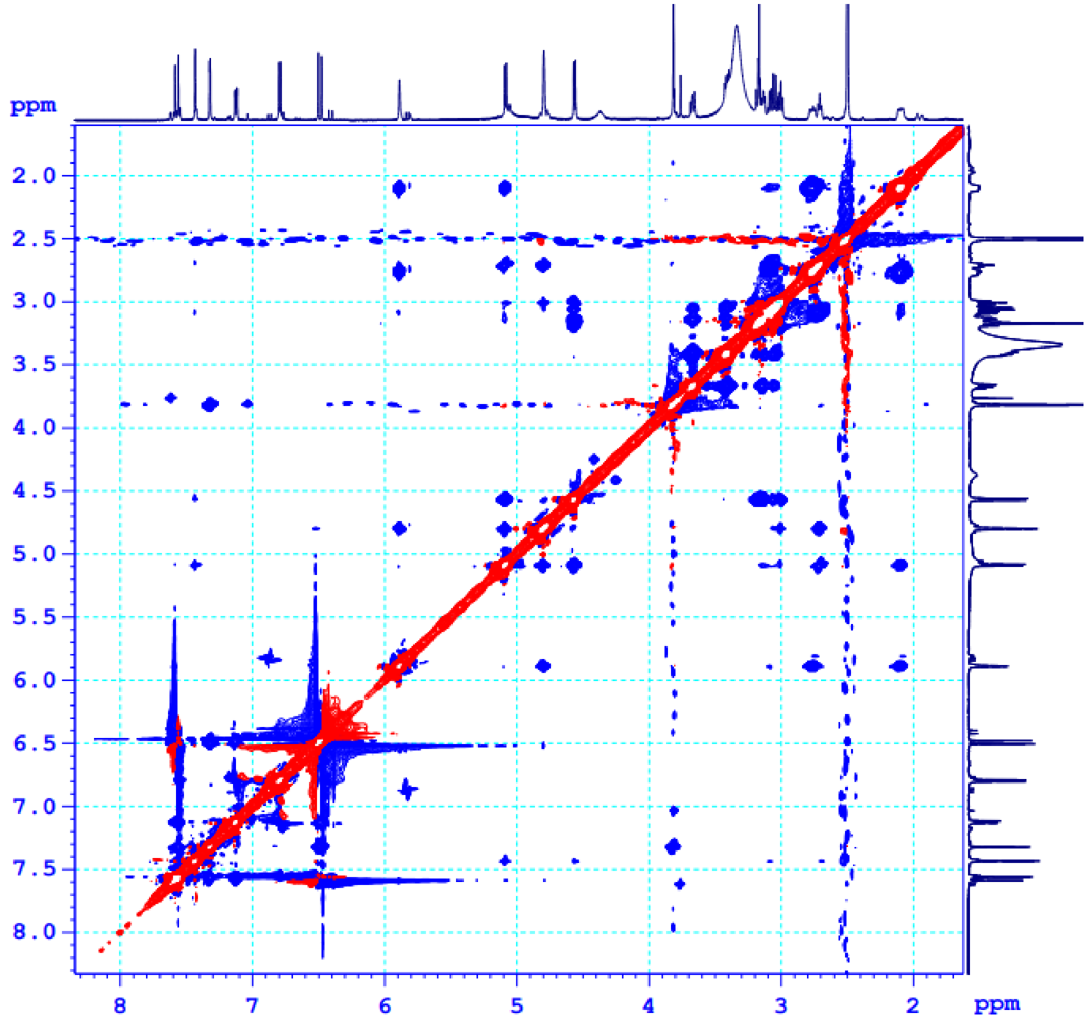

Figure S6. ROESY spectrum of compound **1**

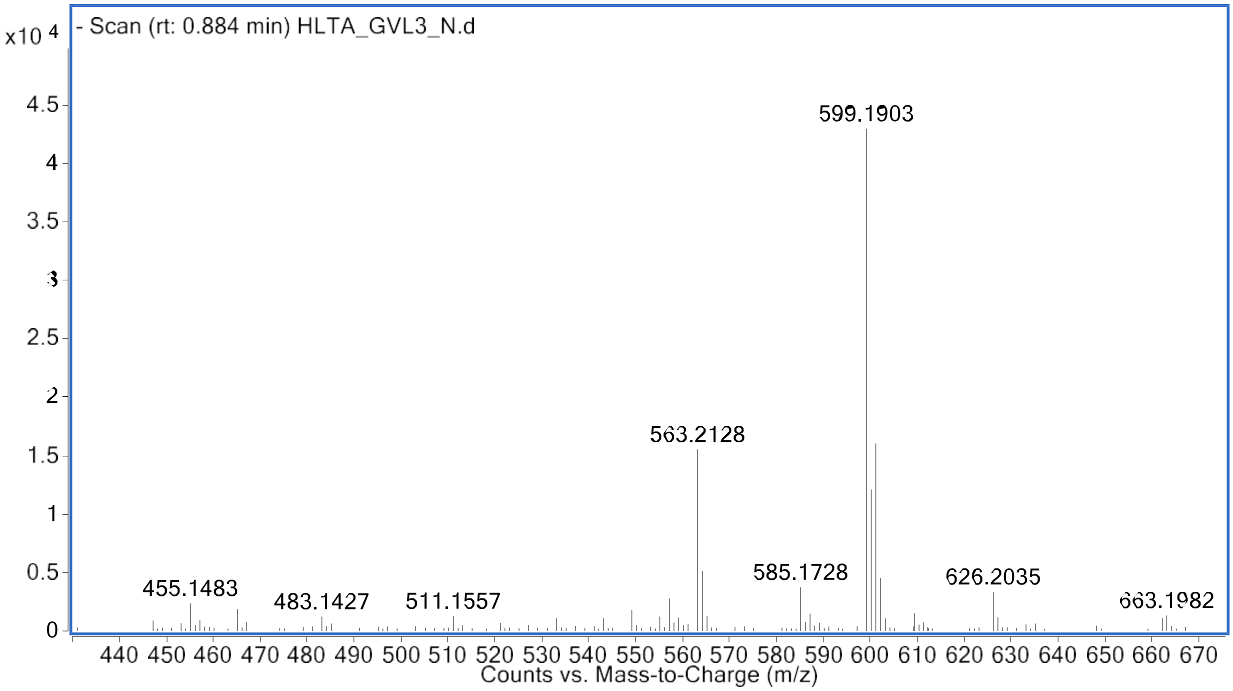

Figure S7. HR-ESI-MS spectrum of compound **1**

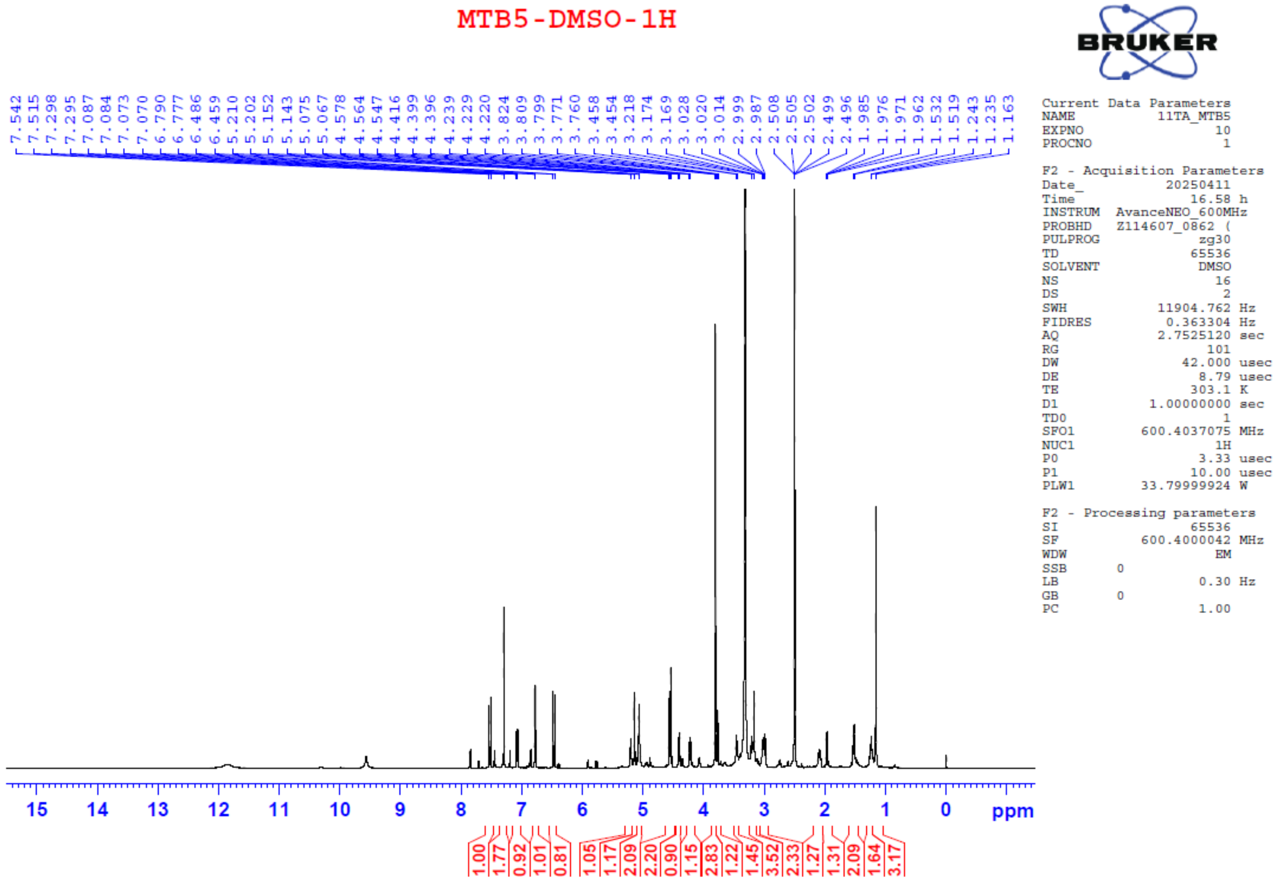

Figure S8. ^{1}H-NMR spectrum of compound **2** and its expanded spectrum

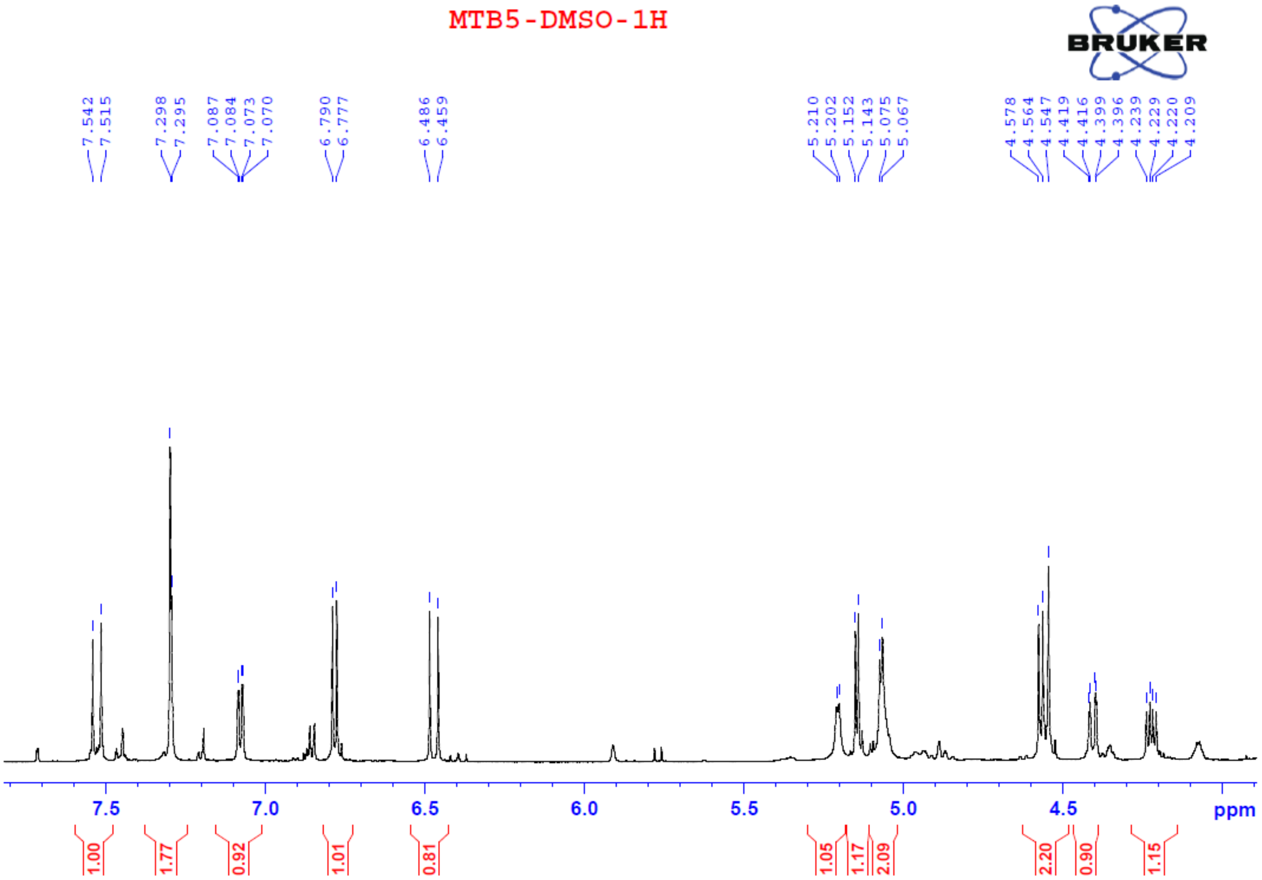
MTB5-DMSO-1H
7.542
7.515
7.298
7.295
7.087
7.084
7.073
7.070
6.790
6.777
6.486
6.459
5.210
5.202
5.152
5.143
5.075
5.067
4.578
4.564
4.547
4.419
4.416
4.399
4.396
4.239
4.229
4.220
4.209
7.5
7.0
6.5
6.0
5.5
5.0
4.5
ppm
1.00
1.77
0.92
1.01
0.81
1.05
1.17
2.09
2.20
0.90
1.15

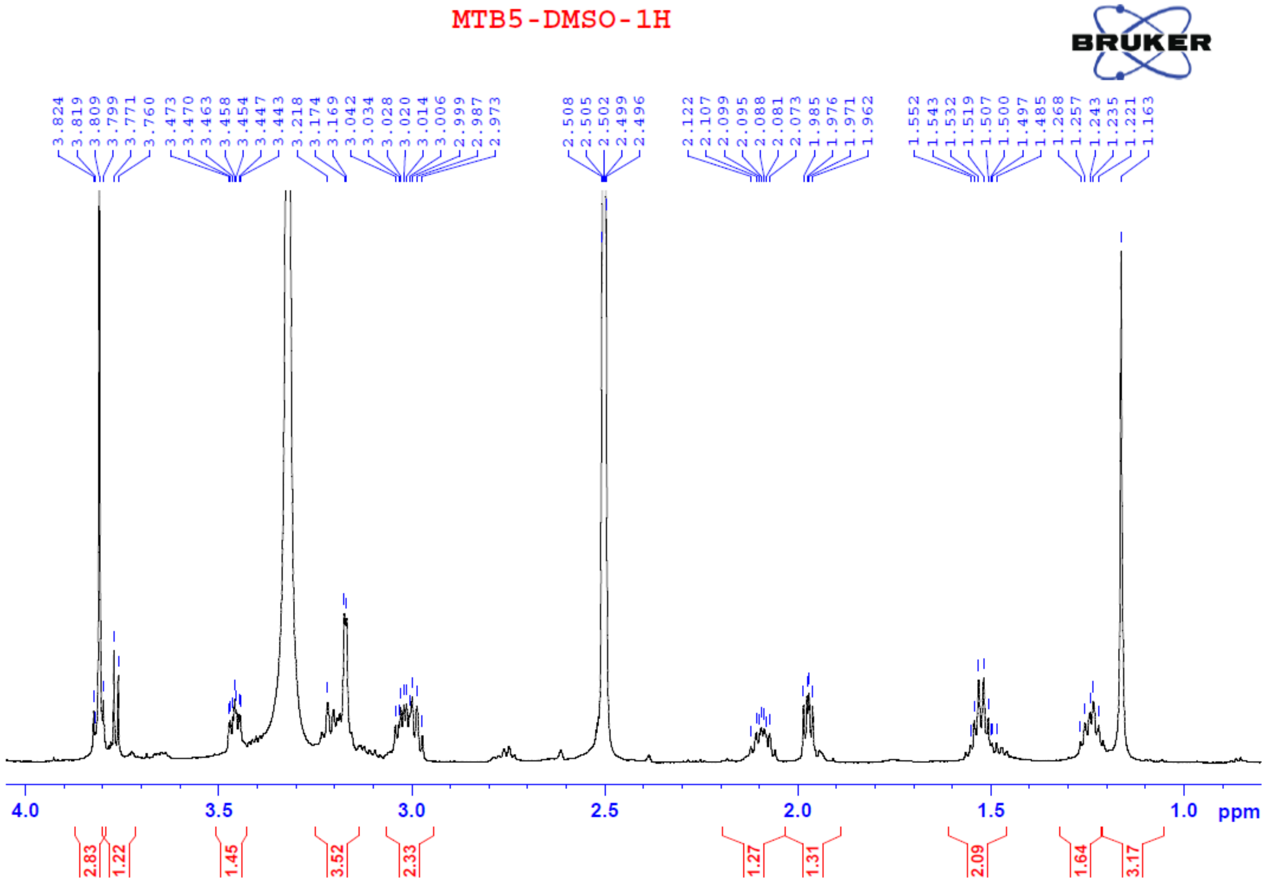
MTB5-DMSO-1H
3.824
3.819
3.809
3.799
3.771
3.760
3.473
3.470
3.463
3.458
3.454
3.447
3.443
3.218
3.174
3.169
3.042
3.034
3.028
3.020
3.014
3.006
2.999
2.987
2.973
2.508
2.505
2.502
2.499
2.496
2.122
2.107
2.099
2.095
2.088
2.081
2.073
1.985
1.976
1.971
1.962
1.552
1.543
1.532
1.519
1.507
1.500
1.497
1.485
1.268
1.257
1.243
1.235
1.221
1.163
4.0
3.5
3.0
2.5
2.0
1.5
1.0
ppm
2.83
1.22
1.45
3.52
2.33
1.27
1.31
2.09
1.64
3.17

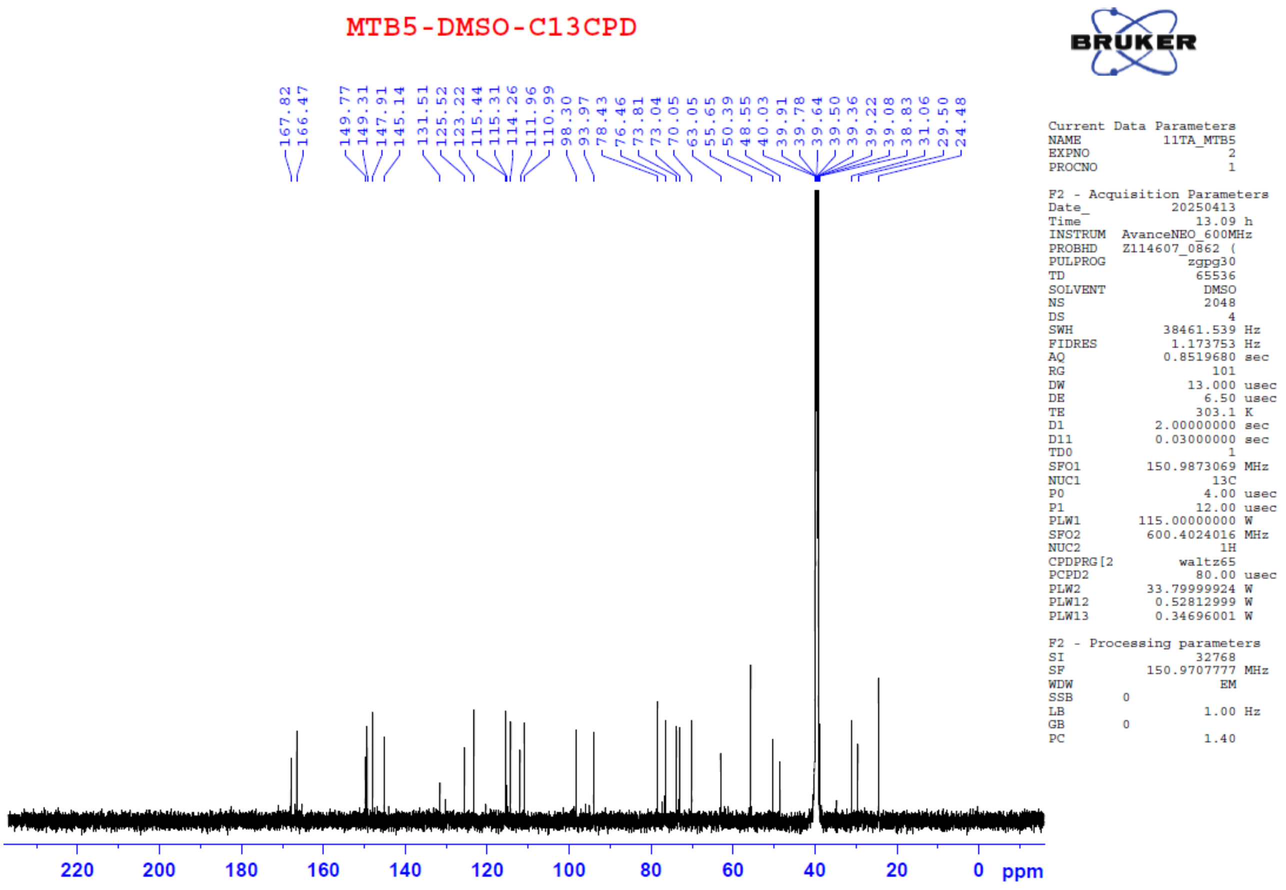

Figure S9. ^{13}C-NMR spectrum of compound **2** and its expanded spectrum

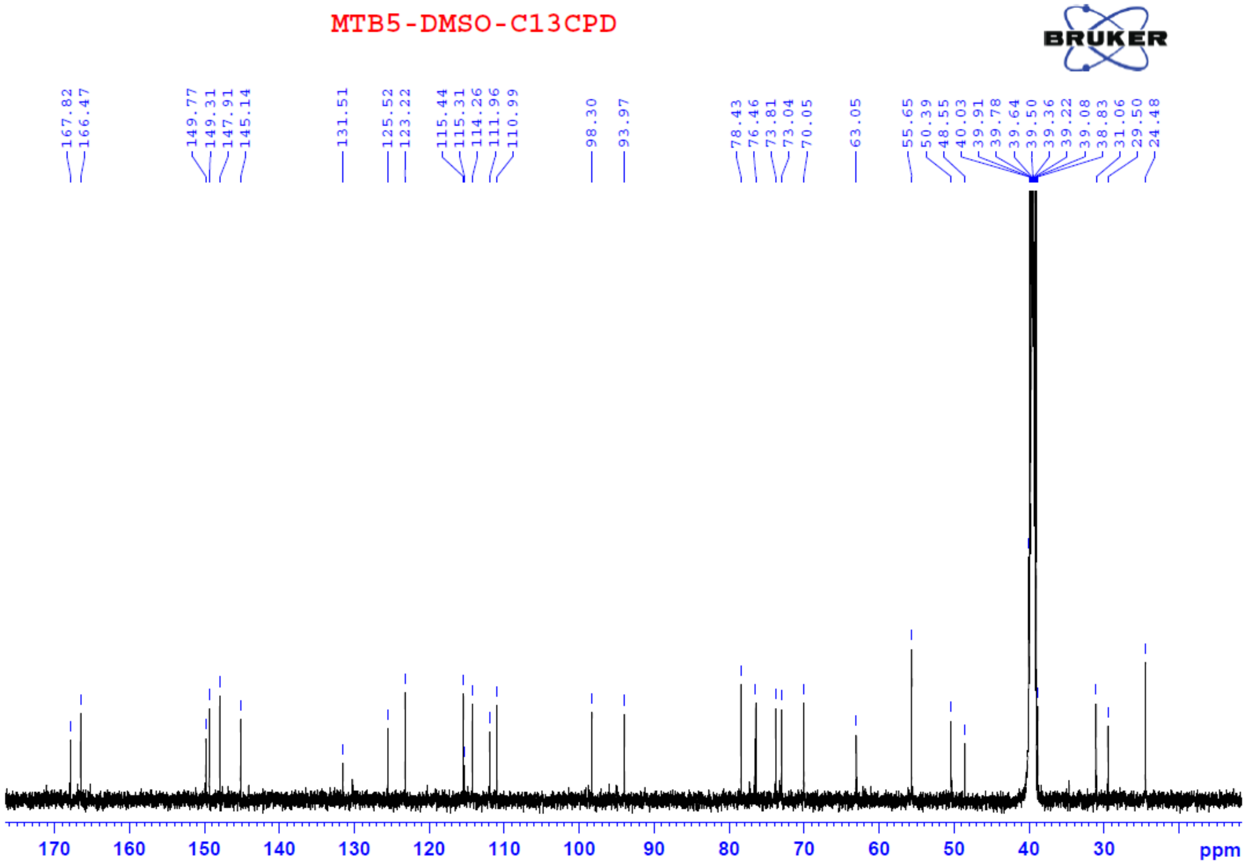
MTB5-DMSO-C13CPD
BRUKER
167.82
166.47
149.77
149.31
147.91
145.14
131.51
125.52
123.22
115.44
115.31
114.26
111.96
110.99
98.30
93.97
78.43
76.46
73.81
73.04
70.05
63.05
55.65
50.39
48.55
40.03
39.91
39.78
39.64
39.50
39.36
39.22
39.08
38.83
31.06
29.50
24.48
170 160 150 140 130 120 110 100 90 80 70 60 50 40 30 ppm

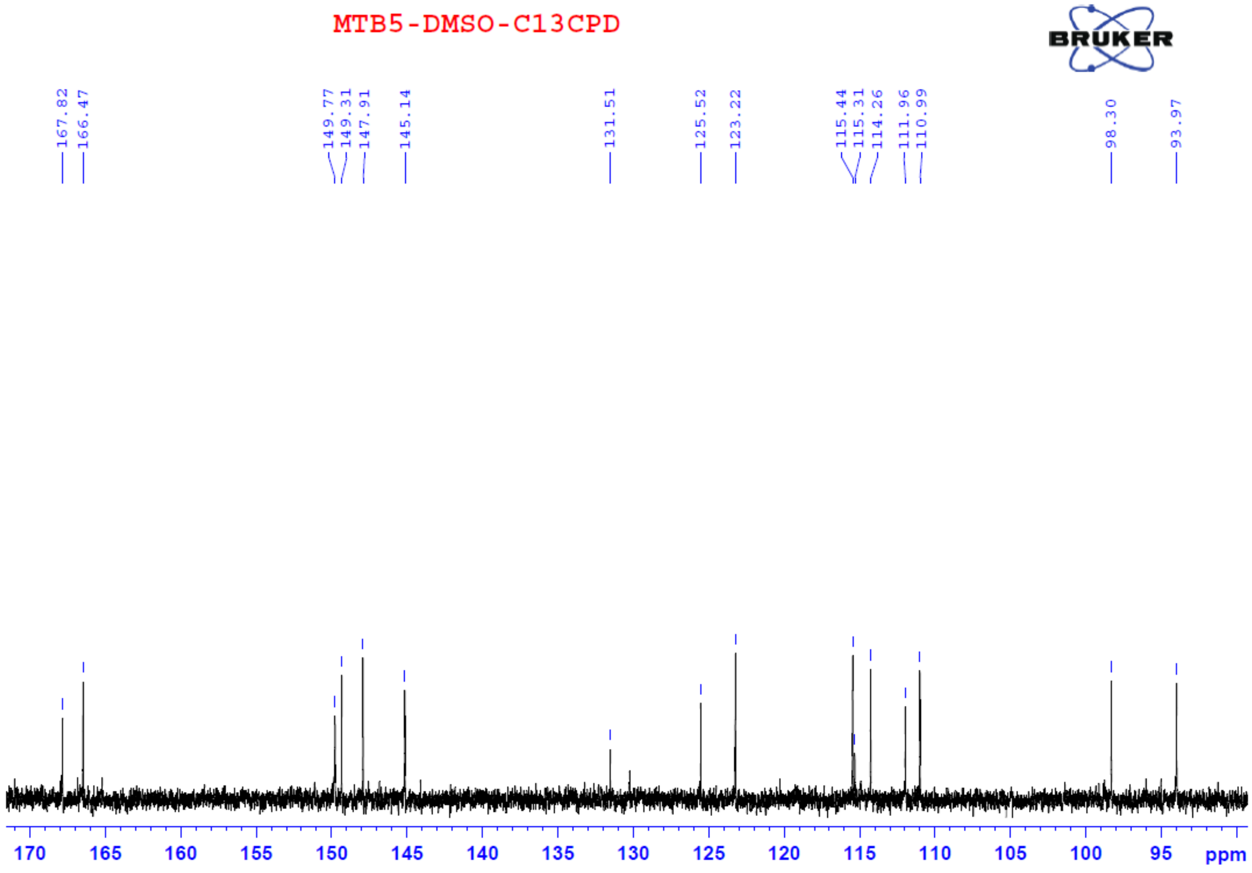
MTB5-DMSO-C13CPD
BRUKER
167.82
166.47
149.77
149.31
147.91
145.14
131.51
125.52
123.22
115.44
115.31
114.26
111.96
110.99
98.30
93.97
170 165 160 155 150 145 140 135 130 125 120 115 110 105 100 95 ppm

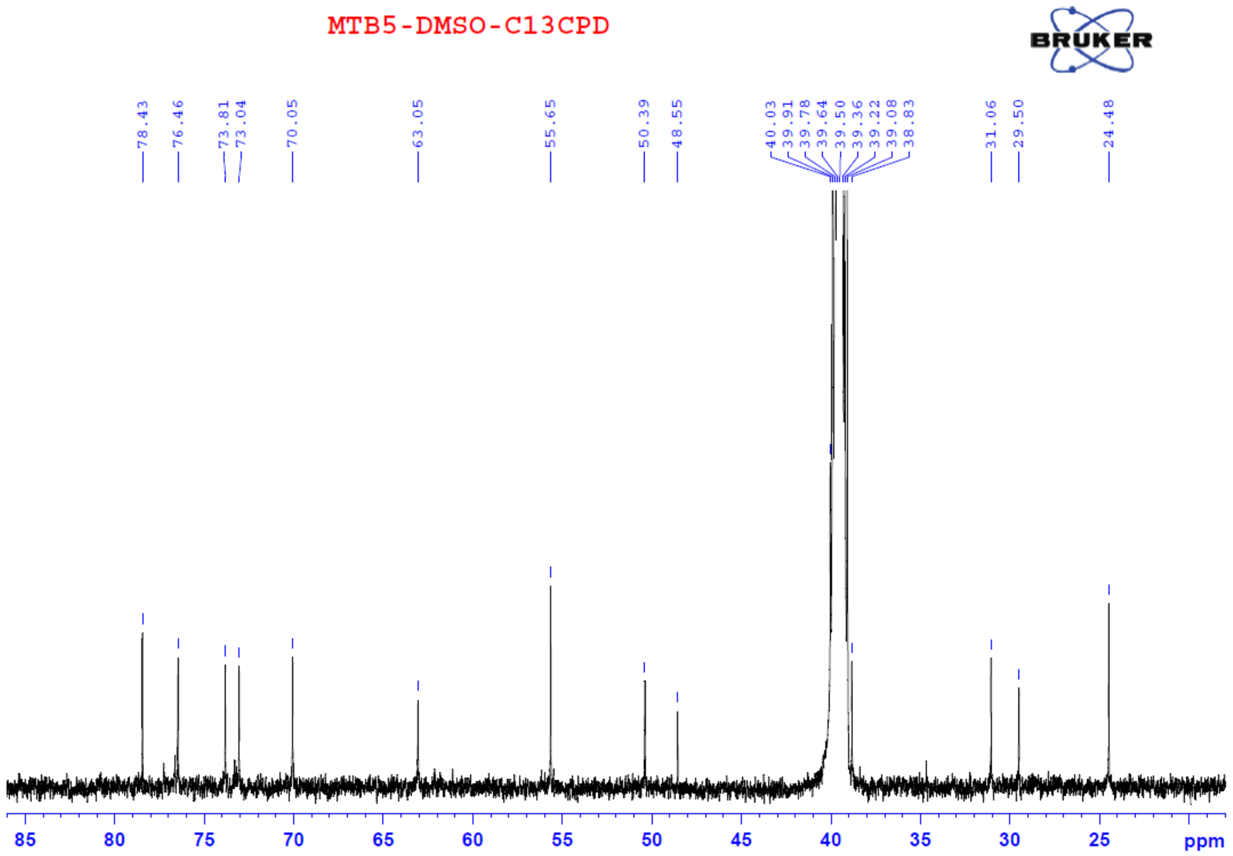
MTB5-DMSO-C13CPD
78.43
76.46
73.81
73.04
70.05
63.05
55.65
50.39
48.55
40.03
39.91
39.78
39.64
39.50
39.36
39.22
39.08
38.83
31.06
29.50
24.48
85
80
75
70
65
60
55
50
45
40
35
30
25
ppm

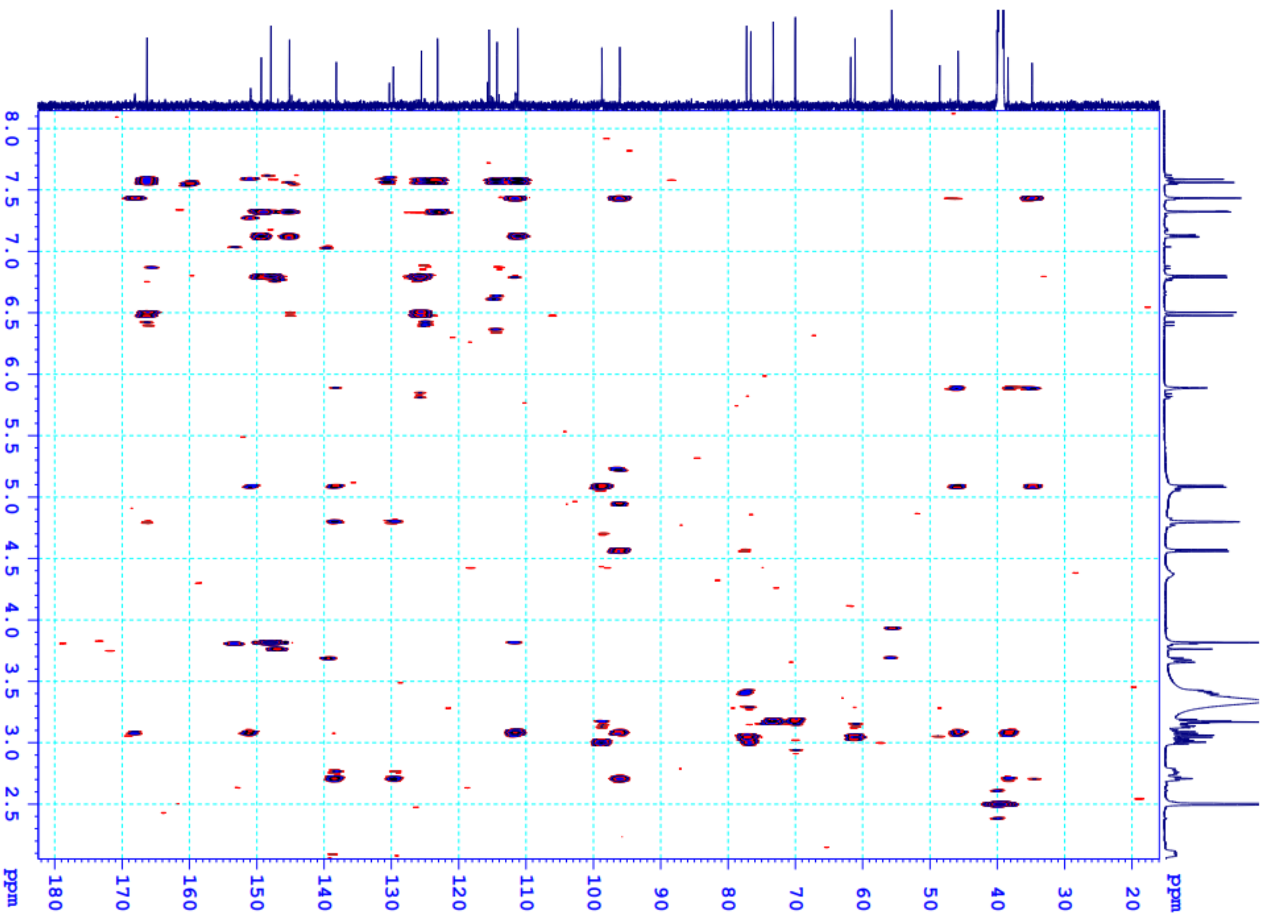

Figure S10. HMBC spectrum of compound **2**

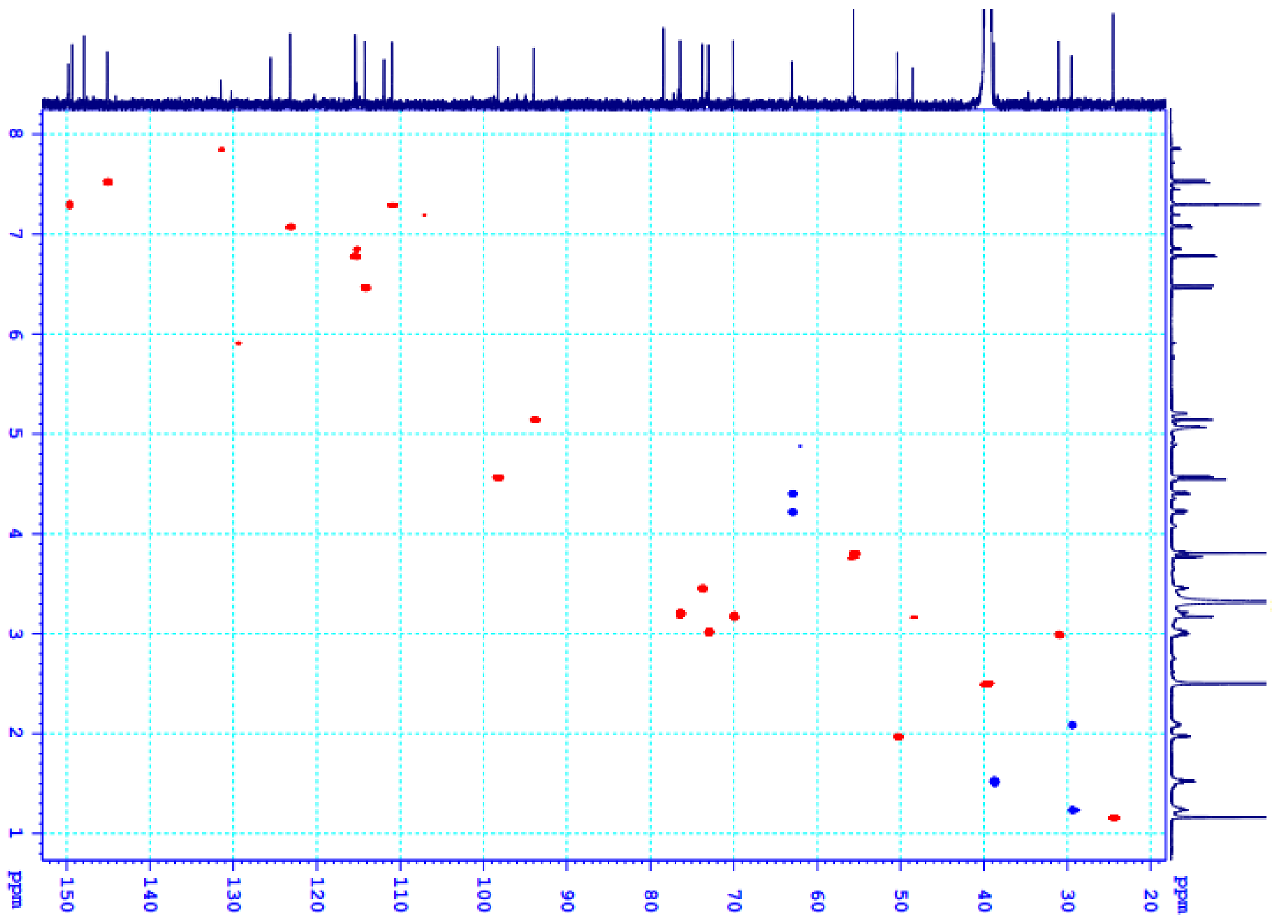

Figure S11. HSQC spectrum of compound **2**

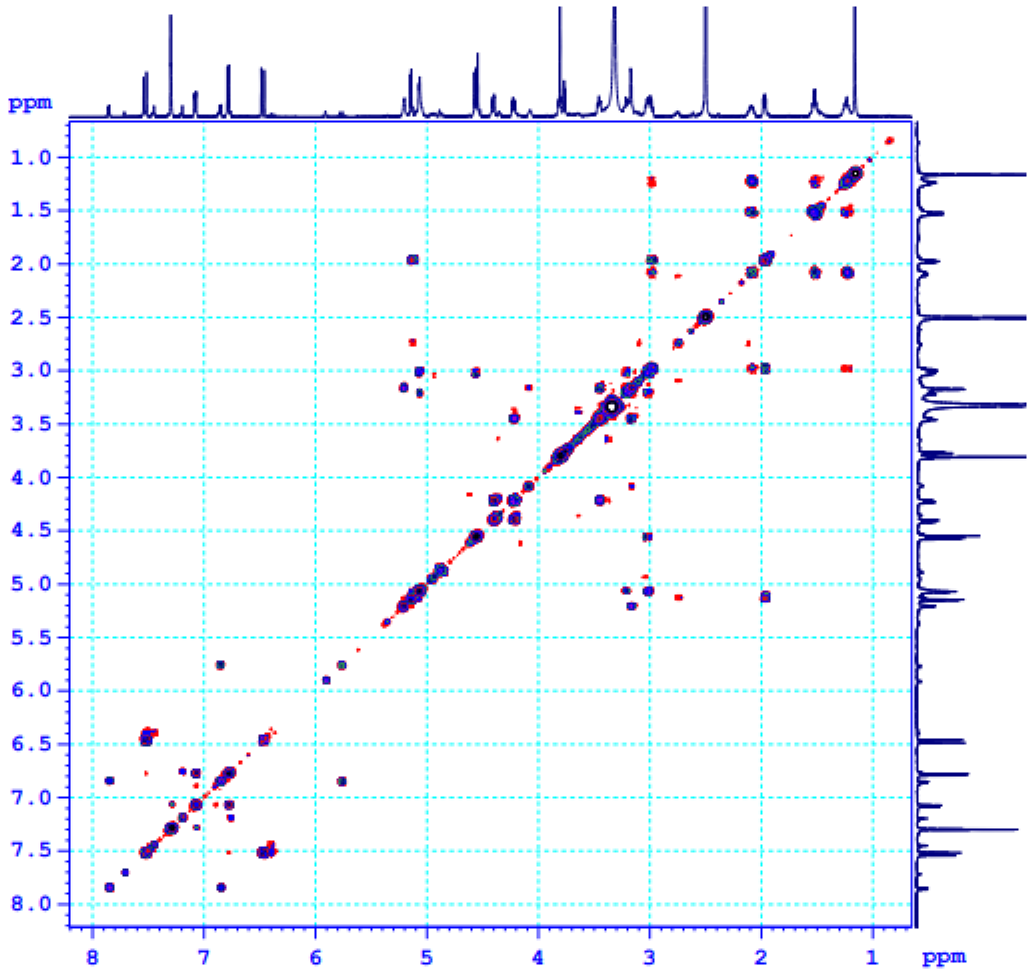

Figure S12. COSY spectrum of compound **2**

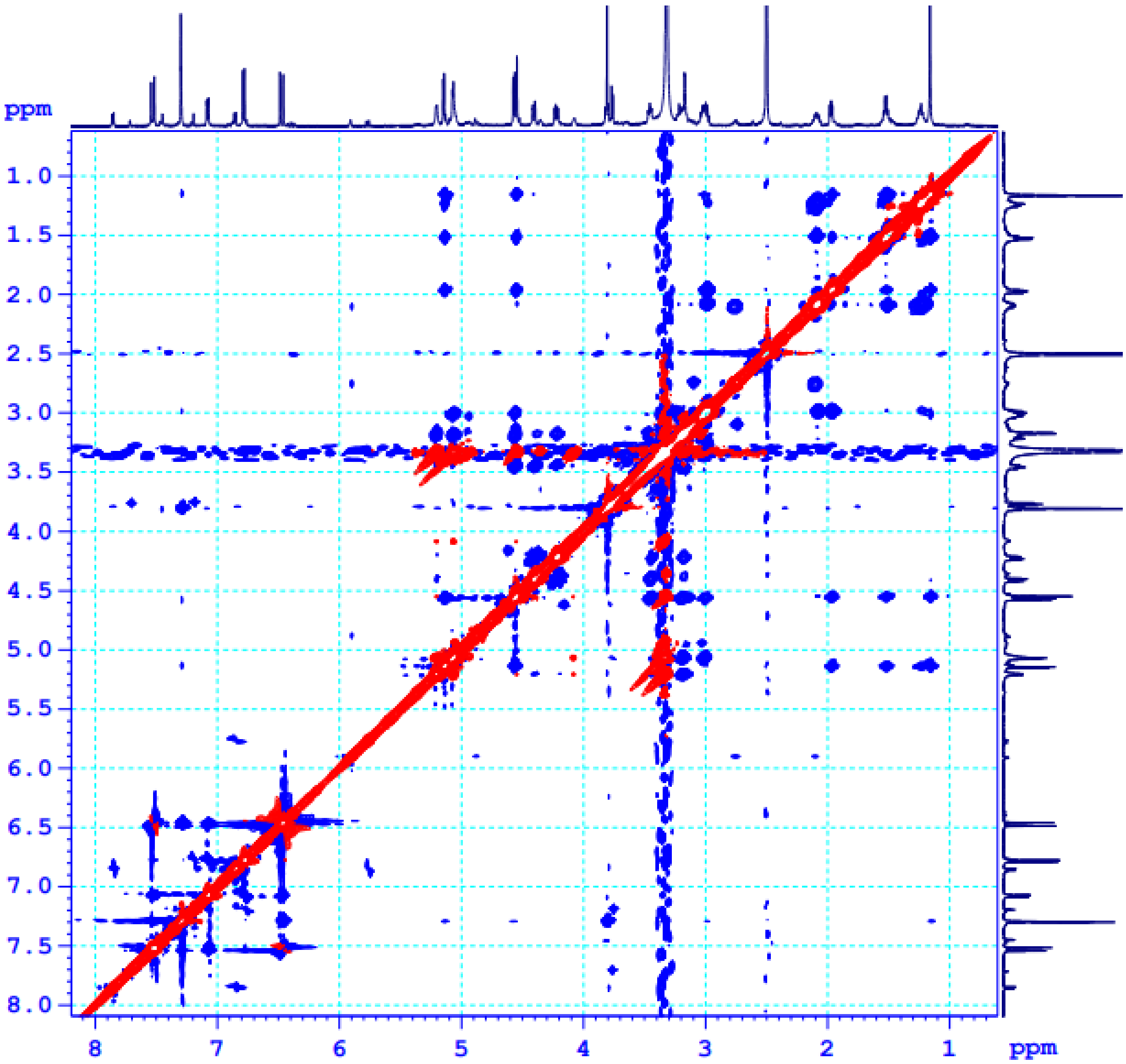

Figure S13. ROESY spectrum of compound **2**

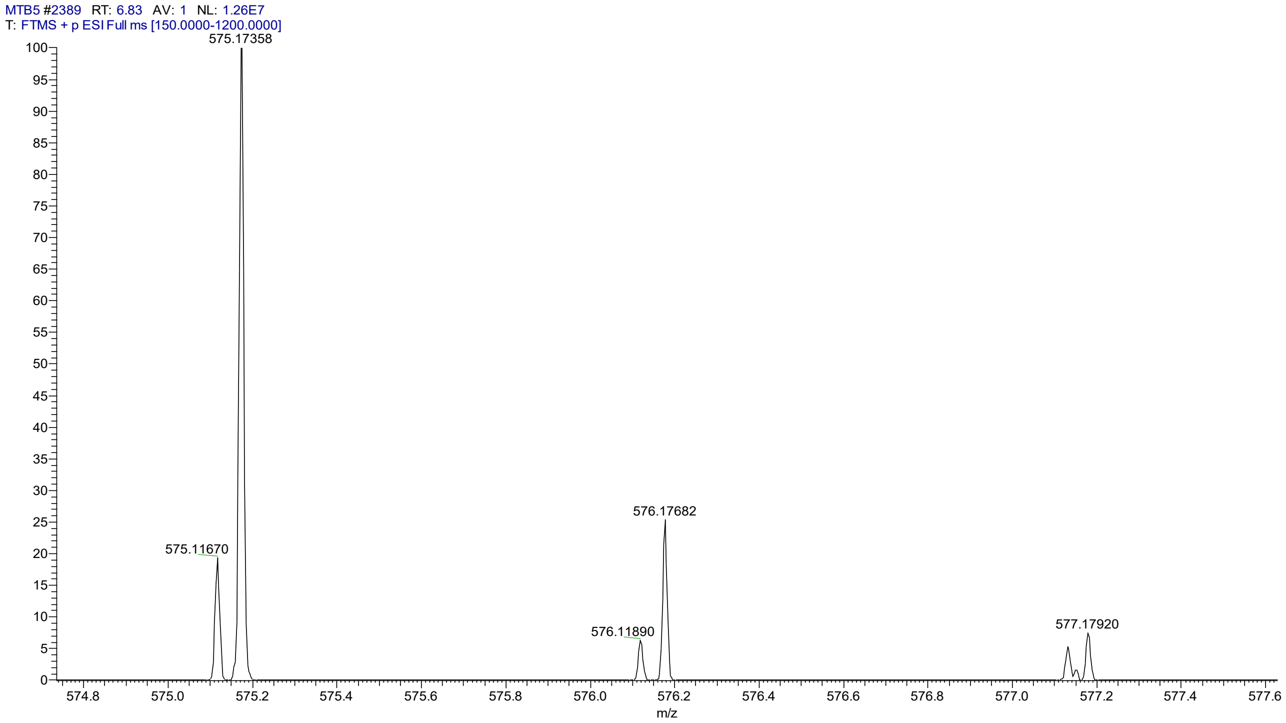

Figure S14. HR-ESI-MS spectrum of compound **2**

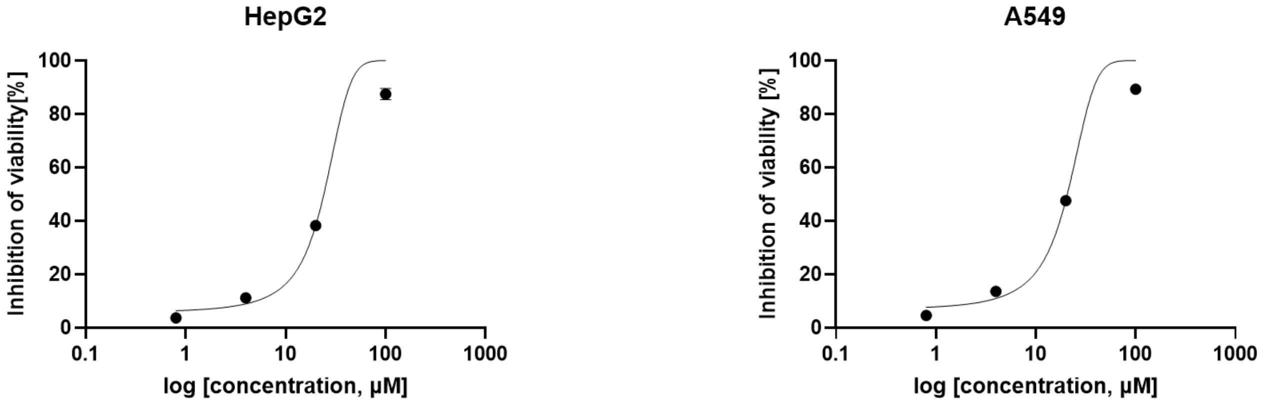

Figure S15. Dose–response curves showing the inhibition of HepG2 and A549 cells by compound 6
